# The Introduction of a Cysteine Residue Modulates The Mechanical Properties of Aromatic‐Based Solid Aggregates and Self‐Supporting Hydrogels

**DOI:** 10.1002/chem.202102007

**Published:** 2021-10-01

**Authors:** Carlo Diaferia, Elisabetta Rosa, Nicole Balasco, Teresa Sibillano, Giancarlo Morelli, Cinzia Giannini, Luigi Vitagliano, Antonella Accardo

**Affiliations:** ^1^ Department of Pharmacy and Research Centre on Bioactive Peptides (CIRPeB) University of Naples “Federico II” Via Mezzocannone 16 Naples 80134 Italy; ^2^ Institute of Biostructures and Bioimaging (IBB), CNR Via Mezzocannone 16 80134 Naples Italy; ^3^ Institute of Crystallography (IC), CNR Via Amendola 122 70126 Bari Italy

**Keywords:** aromatic peptides, cysteine oxidation, hydrogels, peptide materials, self-assembling

## Abstract

Peptide‐based hydrogels, originated by multiscale self‐assembling phenomenon, have been proposed as multivalent tools in different technological areas. Structural studies and molecular dynamics simulations pointed out the capability of completely aromatic peptides to gelificate if hydrophilic and hydrophobic forces are opportunely balanced. Here, the effect produced by the introduction of a Cys residue in the heteroaromatic sequence of (FY)3 and in its PEGylated variant was evaluated. The physicochemical characterization indicates that both FYFCFYF and PEG8‐FYFCFYF are able to self‐assemble in supramolecular nanostructures whose basic cross‐β motif resembles the one detected in the ancestor (FY)3 assemblies. However, gelification occurs only for FYFCFYF at a concentration of 1.5 wt%. After cross‐linking of cysteine residues, the hydrogel undergoes to an improvement of the rigidity compared to the parent (FY)3 assemblies as suggested by the storage modulus (G’) that increases from 970 to 3360 Pa. The mechanical properties of FYFCFYF are compatible with its potential application in bone tissue regeneration. Moreover, the avalaibility of a Cys residue in the middle of the peptide sequence could allow the hydrogel derivatization with targeting moieties or with biologically relevant molecules.

## Graphical abstract

**Hydrogels based** on aromatic peptides can be generated taking advantages from π‐π stacking and hydrogen bondings between the side chain of amino acids. The insertion of a crosslinkable cysteine residue in the middle of the peptide motif could allow the achievement of novel materials with improved mechanical properties for tissue engineering applications.

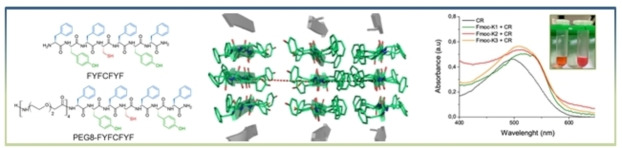

## Introduction

Amino acids and peptides are crucial entities in biological systems, covering functional and structural roles. Due to their biocompatibility, easy accessibility and well‐known chemistry, they have been widely used as constitutive and versatile elements for the generation of supramolecular architectures, including nanofibers, nanodots, plates, film and hydrogels (HGs).^[1]^ These latter materials drew increasing interest during the last decade due to their large range of applications.^[2]^ For instance, they have found use as drug reservoir,^[3]^ healthcare tools^[4]^ and scaffolds for tissue engineering.^[5]^ Peptides based HGs share a common gelation mechanism, driven by physical and no‐covalent cross linking network formation in which monomers mutually interact via aromatic stacking, hydrogen bonding and water exclusion effect. As consequence of these molecular interactions, solvated amyloid‐like fibrils are formed. The subsequent complex fibrils entanglement promotes the formation of a tridimensional supramolecular network that generates the gel, restricting flow of solvent and imprinting the non‐Newtonian behaviour of the final material. Fmoc N‐terminally protected mono‐, di‐ and tripeptides and peptide amphiphiles (PAs) are some examples of peptide monomers used as constitutive elements for HGs matrices generation.^[6]^ These monomers can differently arrange in well‐known structural motifs, for example β‐hairpin, β‐sheet and α‐helix. It was observed that the appropriate modulation of the peptide primary sequence can allow controlling the morphology, the structural and the functional properties of the hydrogel.^[6]^ One of the most used strategy consists in using primary sequences containing an alternation of charged/apolar amino acids.^[7]^ In these peptide sequences, HGs formation can be prompted by the protonation/deprotonation state of charged amino acids (such as Lys, Arg, Glu or Asp). Structurally, it was recently observed that the presence of negatively or positively charged amino acids in the peptide sequence, characterized by an alternation of polar and apolar residues, is not essential for aggregation and gelation.^[8]^ Noteworthy examples in this context are represented by the peptides PEG8‐(FY)3^[9]^ and by its analogues (Nal‐Dopa)3, (Nal‐Y)3, (F‐Dopa)3 and PEG8‐(Nal‐Y)3 [where Dopa= dopamine, Nal= naphthylalanine, PEG= polyethylene glycol], which are able to self‐assemble in self‐supporting hydrogels at a concentration of 1.0 % wt.^[10]^ The structural characterization of the resulting HGs highlighted the organization in β‐sheet fibrillary networks with an antiparallel orientation of peptide sequences. Wide angle X‐ray scattering (WAXS) data, collected on solid fibers of (FY)3, combined with molecular dynamics (MD) simulations studies allowed to identify the essential structural elements that are at the basis of the assembly. The proposed model indicates the presence of two well distinct interfaces: a hydrophobic and highly rigid one generated by the interactions between phenyl rings of Phe residues, and a hydrophilic one caused by interacting Tyr side chains of facing strands.^[9]^ Analogously, peptide derivatives, in which Phe and Tyr aromatic residues are replaced with non‐coded ones like Nal and Dopa, exhibit propensity to gelificate under specific experimental conditions. The derivatization of this class of peptides with a PEG moiety was found to cause an increase or a decrease of their propensity to gelificate.^[10]^

This result suggests the key role played by the hydrophilic/hydrophobic balance in the gel formation. In order to further investigate this class of aromatic peptide hydrogelators, we designed a novel peptide analogue in which the (FY)3 motif was modified into a heptapeptide sequence, inserting a cysteine residue (C) in the middle of its primary sequence. Moreover, to elucidate the effect of the PEG decoration on the aggregation behaviour, the resulting peptide FYFCFYF was also derivatized with a monodisperse PEG moiety containing eight oxoethylenic units (PEG8‐FYFCFYF). Due to its thiol group, the cysteine residue may be chemically treated to prompt cross‐link in peptidic and polymeric hydrogels. Aggregation properties of these Cys‐containing peptides were fully investigated using a set of biophysical techniques. Gelification features were analysed and mechanical properties of the resulting gels, before and after cross‐linking, were also characterized by rheology.

## Results and Discussion

**Figure 1:**
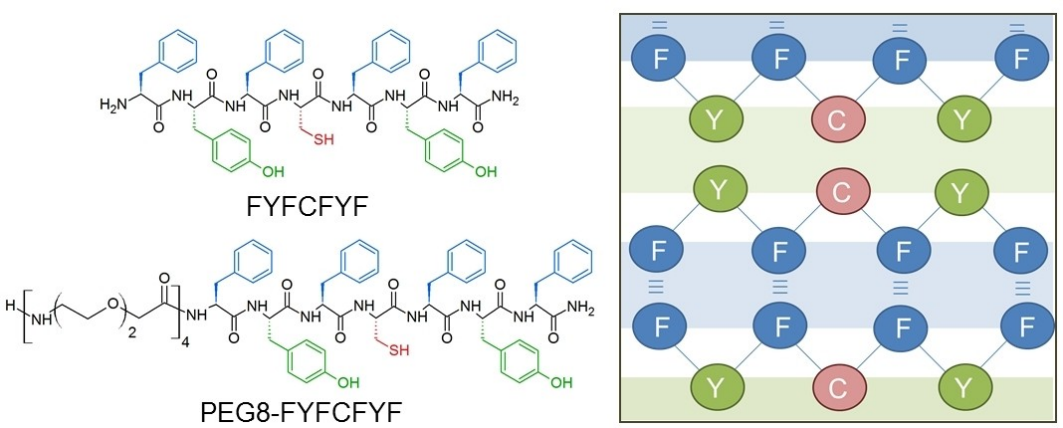
Chemical structure of FYFCFYF and PEG8‐FYFCFYF cysteine containing peptides. Schematic representation of the double interfaces supposed organization: green region indicates the hydrophilic interface; blue one identifies the hydrophobic interface.

Chemical formulas of the peptide FYFCFYF and of its PEGylated variant (PEG8‐FYFCFYF) characterized in the present study are reported in Figure 1. Both peptides were designed modifying the (FY)3 sequence, by inserting a cysteine residue (C) in the middle of the heteroaromatic sequence. The position of the Cys amino acid was chosen in order to generate a symmetrical disposition of the aromatic residues and to locate it into the structural centre of the resulting nanostructures (Figure 1). With respect to other polar neutral residues (e. g. serine or glutamine), the Cys modification makes possible the generation of additional chemical cross linking via thiol (SH) oxidation. This approach allows the introduction of a tunable and reversible functionality without a radical modification of the non‐covalent pathway network. The Cys‐Cys disulfide bridge formation was scrutinized also in its impact on the mechanical properties of the biomaterials formed by the proposed peptides.

## Molecular modelling and dynamics

**Figure 2:**
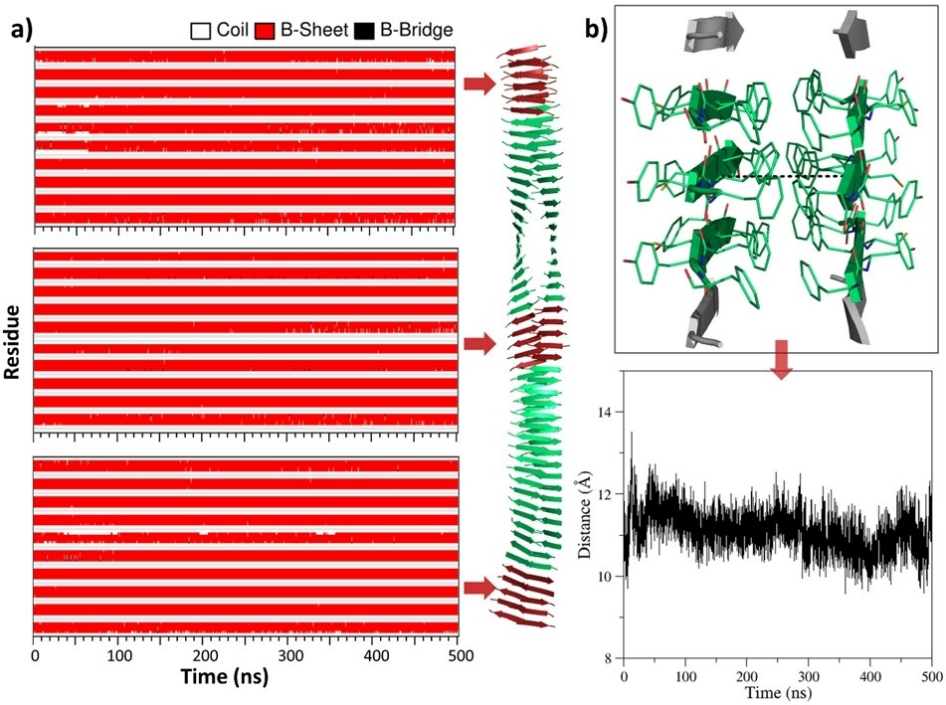
a) Time evolution of secondary structure in the MD simulation carried out starting from the flat model of FYFCFYF_ST50_SH2. Secondary structure is reported only for the strands belonging to the central region and to both terminal ends that are shown in red in the average structure computed in the trajectory region 250–500 ns. b) Time evolution of the distance between two representative C^α^ atoms of the facing sheets along the MD trajectory.

In order to gain insights into the aggregation propensity of FYFCFYF, we preliminary performed computational studies on the putative assemblies formed by this peptide. A molecular model of the peptide FYFCFYF, which also constitutes the structural spine of PEG8‐FYFCFYF, was generated by exploiting the information derived from the structural characterization of the related (FY)3 peptide assemblies.[^9^, ^11^] In particular, an individual β‐sheet was generated by assuming an antiparallel association of their β‐strands. The alternative orientation between the consecutive residues within the strands produces in the β‐sheet an apolar and a polar surface made by either Phe or Tyr/Cys side chains, respectively. The juxtaposition of two β‐sheets, through the association of their apolar surfaces, generated a potential basic element of the FYFCFYF system. The three‐dimensional representation of this starting model, containing two fifty‐stranded β‐sheets (FYFCFYF_ST50_SH2), is illustrated in Figure S1a. The stability and the dynamics of this model was evaluated by MD simulations. A 500 ns simulation of the FYFCFYF_ST50_SH2 system indicates that the initial model undergoes a significant structural rearrangement. As indicated by the gyration radius and by the root mean square deviation (RMSD) values of the trajectory structures *versus* the initial model, an equilibrated region is reached only after 250 ns of the simulation (Figures S2a and S2b). Although the secondary β‐structure of the assembly is well preserved in the entire simulation timescale, a clear twisting of the two‐sheet model is observed (Figure 2a). Notably, the inter‐sheet distance (∼11 Å) is also well preserved in the simulation, further

confirming the stability and the relative rigidity of the assembly (Figure 2b). The inspection of the side chain flexibility (Figure S3a) and their rotameric states (Figure S3b) provides insights into the basis of the particular stability and rigidity of the assembly. Indeed, recurrent and preserved rotameric states are detected for the inter‐strand interacting side chains with the alternation of strands with residues either in the trans or gauche χ1 state. The alternation of the side chains also assures the interactions that stabilized the hydrophobic interface.

**Figure 3:**
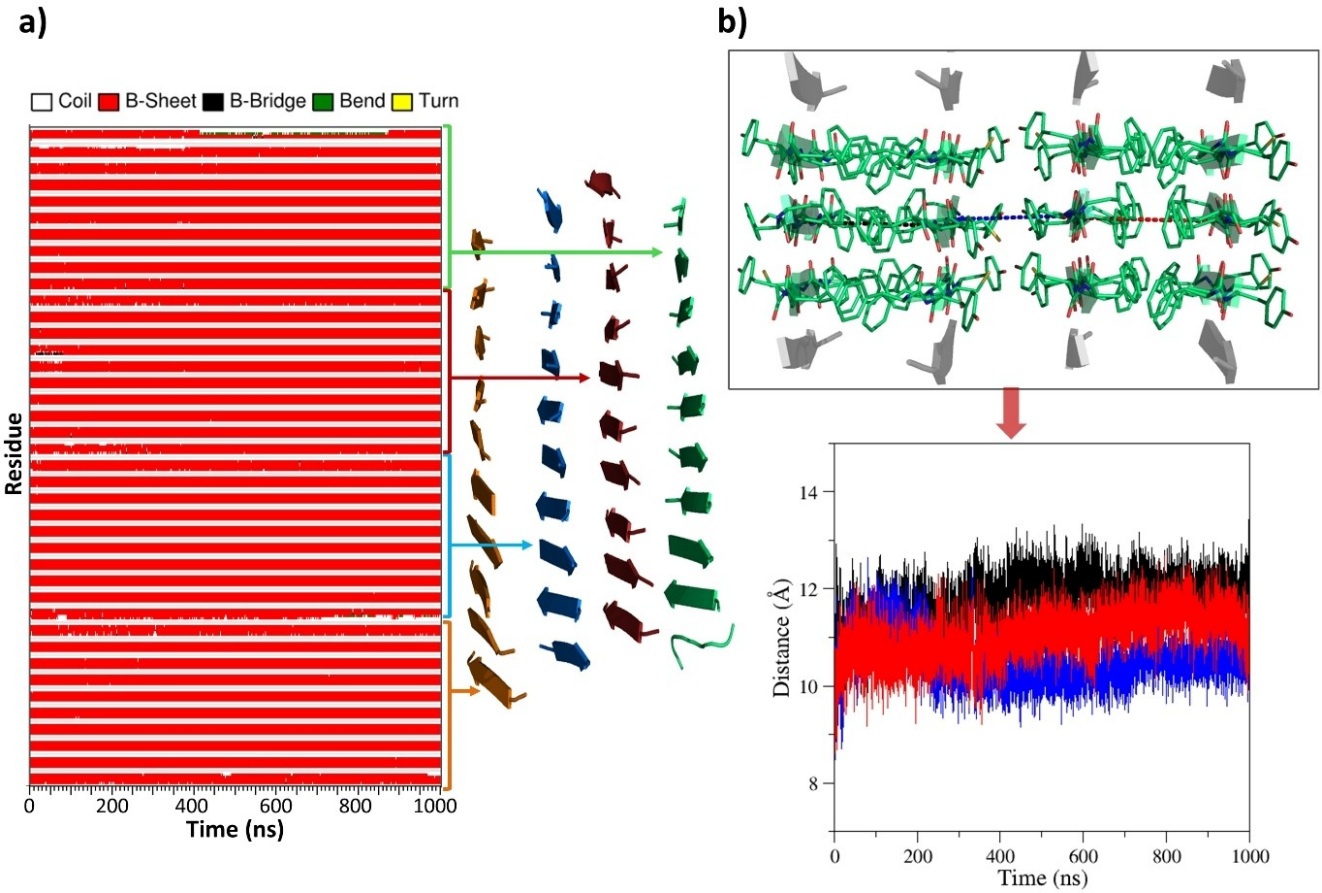
a) Time evolution of secondary structure in the MD simulation carried out starting from the flat model of FYFCFYF_ST10_SH4. The average structure computed in the trajectory region 500–1000 ns is also shown. b) Time evolution of the distances between two representative Cα atoms of the facing sheets along the MD trajectory.

These analyses were expanded by considering an assembly formed by four ten‐stranded β‐sheets (see Methods for details) containing both hydrophobic and hydrophilic interfaces (FYFCFYF_ST10_SH4, Figure S1b). The MD characterization of FYFCFYF_ST10_SH4 clearly indicates that the stability detected for FYFCFYF_ST50_SH2 extends to larger assemblies without being significantly affected by the presence of a hydrophilic interface (Figures S4). As shown in Figure 3b, the inter‐sheet distance found for the hydrophilic interface resembles that detected for the hydrophobic ones (∼11 Å). As observed for FYFCFYF_ST50_SH2, also the side chains of FYFCFYF_ST10_SH4 are endowed with a remarkable rigidity (Figures S5a and S5b). This is particularly evident for the internal β‐sheets within this four‐sheet model. The inspection of the distances between the side chains of the Cys residues located in facing sheets within the hydrophilic interface indicates that the sulphur atoms may come as close as 3.5 Å, the minimal distance expected on the basis of their van der Waals radius (∼1.8 Å) (Figure S6).

**Figure 4:**
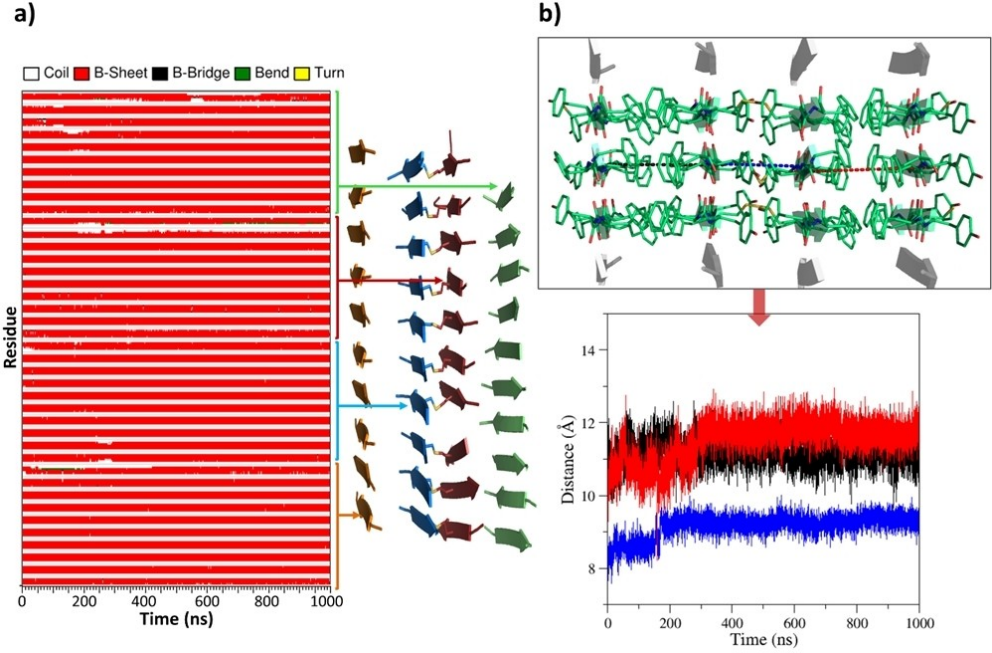
a) Time evolution of secondary structure in the MD simulation carried out starting from the flat model of FYFCFYF_ST10_SH4 SS. The average structure computed in the trajectory region 500–1000 ns is also shown. b) Time evolution of the distances between two representative C^α^ atoms of the facing sheets along the MD trajectoryi.

Based on this observation, we verified the possibility of having Cys residues in their oxidized state in this assembly. To this aim, we considered a variant of FYFCFYF_ST10_SH4 in which the Cys residues at the hydrophilic interface were involved in the formation of Cys‐Cys disulphide bonds (FYFCFYF_ST10_SH4 SS) (Figure S1c). A one μs MD simulation clearly indicates that the presence of the disulphide bridge is fully compatible with the formation of β‐rich assemblies of FYFCFYF (Figures S7 and S4). Also, the inter‐sheet distances detected in FYFCFYF_ST10_SH4 SS are very similar to those detected in FYFCFYF_ST10_SH4 (Figure 4).

Collectively, the present findings indicate that FYFCFYF peptide forms pretty stable β‐sheet assemblies in which both the hydrophobic and the hydrophilic interface are rather rigid. These assemblies may be formed with the Cys residues either in its reduced or oxidized state.

## Peptide synthesis and aggregation

Both the peptide derivatives were synthetized using solid phase peptide synthesis protocols (SPPS) with Fmoc/OtBu strategy. After their purification by RP‐HPLC chromatography, peptides were characterized by ESI mass spectrometry (see Figure S8). The log*P* values, estimated using the ACD Lab ChemSketch software, predict the intrinsic water solubility of peptides. Due to the presence of the ethoxylic moieties, PEG8‐FYFCFYF (log*P*=3.57±1.05) shows a 40‐fold higher water solubility (∼10.0 mg/mL) compared to FYFCFYF (log*P*=7.57±0.92; water solubility of 0.240 mg/mL). A diametric behaviour was noted using the organic solvent 1,1,1,3,3,3‐hexafluoro‐isopropan‐2‐ol (HFIP).[^11a^, ^12^] Indeed, in HFIP the peptide FYFCFYF is highly soluble (up to 200 mg/mL), whereas PEG8‐FYFCFYF is moderately soluble (up to 5 mg/mL). According to their different solubility, peptide solutions were prepared using two approaches: PEG8‐FYFCFYF solutions were prepared by a direct dissolution of the lyophilized powder in water, whereas FYFCFYF ones were obtained by dilution in water of a HFIP stock solution (100 mg/mL). Then, the organic solvent was removed using N2 flow. Peptide concentration was then analytically quantified using UV‐Vis spectroscopy. Preliminarily, we evaluated the capability of both the peptides to aggregate in solution (at 2.0 mg/mL) using the Dynamic Light Scattering (DLS) technique. As expected, DLS profiles of both Cys‐containing peptides exhibit a monomodal distribution (see Figure S9a), with a mean diameter of 164 and 197 nm for PEGylated and unPEGylated derivatives, respectively. This result indicates that aggregation phenomena occur in solution at the investigated concentrations. The formation of nanoarchitectures was further investigated by fluorescence spectroscopy. Despite the presence of four Phe residues in the peptides, their self‐assembling propensity cannot be evaluated by simply considering the Phe excimer formation. Indeed, from the inspection of Figure S9b, it can be observed that a FRET (Förster resonance energy transfer) phenomenon occurs between the aromatic

chains of Phe and Tyr residues, which work as the donor and the acceptor, respectively.The evidence of the FRET phenomenon is well proved by the superimposition of the emission profiles recorded for PEG‐FYFCFYF (0.005 mg/mL) upon excitation at 257 and 275 nm, which correspond to the absorbance wavelength of Phe and Tyr, respectively. Hence, fluorescence spectra of peptide solutions at different concentrations were acquired exciting samples at the wavelength of the Tyr. From the inspection of the spectra, it can be observed an emission peak at 305 nm, whose intensity decreases upon increasing the concentration (Figure S10a). This behaviour, due to quenching phenomena occurring at high peptide concentration, was previously observed for other aromatic peptides such as PEGylated and unPEGylated (FY)3, Y4 and F6 derivatives.[^9^, ^11b^, ^13^] By further diluting the peptide solution below 0.05 mg/mL, we also observed the appearance of another more intense peak centred at 340 nm (Figure S10b). The presence of this additional peak suggests the existence of different equilibria in solution.

**Figure 5:**
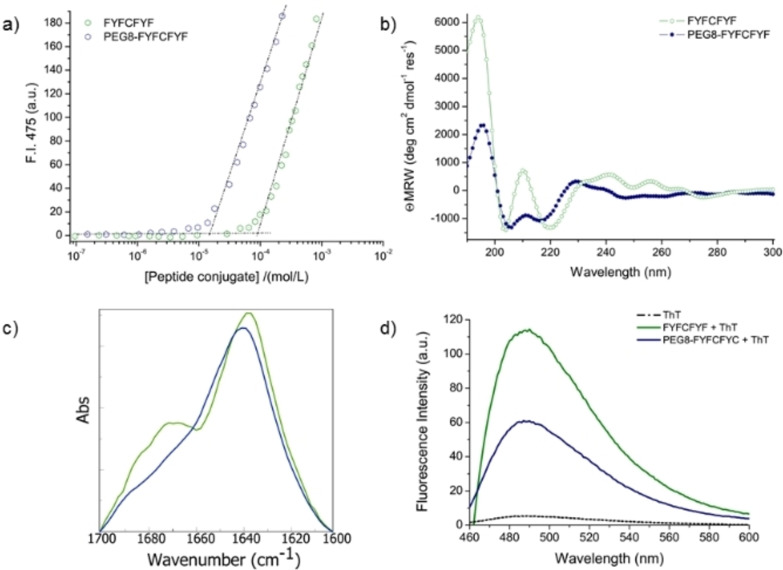
Characterization of peptide aggregates in solution. a) Fluorescence intensity of the ANS fluorophore at 475 nm *versus* the concentration of each peptide. CAC values are calculated from the visual break points. b) CD spectra of peptide solutions at a concentration of 1.0 mg/mL. All the spectra are recorded between 300 and 190 nm. c) Absorbance deconvolution of FTIR spectra in the amide I region for FYFCFYF and PEG8‐FYFCFYF. d) Fluorescence spectra of ThT coincubated with each peptide derivative after the subtraction of the peptide self‐fluorescence. The spectrum of ThT alone is also reported for comparison.

A more accurate determination of the critical aggregation concentration (CAC) was achieved by fluorescence titration method previously used also for micelles^[14]^ or self‐assembled peptide aggregates.^[15]^ CAC values are graphically extrapolated in the break point of the titration graph, this latter obtained plotting the fluorescence intensity of ANS in the maximum at 475 nm as function of the peptide concentration. CAC evaluation experiments for both Cys‐containing peptides are reported in Figure 5a, and the values are 9.09 ⋅ 10^−5^ mol/L (94.1 μg/mL) and 1.50 ⋅ 10^−5^ mol/L (24.2 μg/mL) for FYFCFYF and PEG8‐FYFCFYF, respectively. A similar trend in the CAC values was previously observed for the PEG8‐(FY)3/(FY)3 sequences, which had a CAC value of 1.50 ⋅ 10^−5^ and 5.98 ⋅ 10^−5^ mol/L.^[9]^ This consideration points out the importance of the PEG moiety in the promotion of the molecular interactions that drive the aggregation mechanism. It is worth noting that the CAC value of PEG8‐FYFCFYF corresponds exactly to the CAC of PEG8‐(FY)3, thus suggesting that the insertion of a Cys residue at the centre of the peptide primary structure does not affect the aggregation propensity of the PEGylated variant. On the other hand, a slight increase of the CAC value can be noticed for FYFCFYF respect to (FY)3. This increase could be probably attributed to an interference of the Cys residue with the mechanism of hydrophobic collapse driving the self‐assembling phenomenon.

## Secondary structure characterization

In order to characterize and analyse the supramolecular behaviour of PEG8‐FYFCFYF and FYFCFYF assemblies, spectroscopic techniques such as Circular Dichroism (CD) and FTIR were used.^[16]^ A CD spectrum covers both the far and the near UV region, where the spectrum is dominated by the amide chromophores and lateral chains of aromatic residues and cysteine absorbance, respectively. CD spectra of both Cys‐containing peptide samples at 1.0 mg/mL were recorded between 190 and 300 nm (see Figure 5b). Differing only for the N‐terminus PEGylation, both CD spectra share a common profile, with a maximum at 195 nm (indicative of π→π* transitions), a relative maximum at 210 nm and two minima located at ∼204 nm and at 221 or 217 nm for FYFCFYF and PEG8‐FYFCFYF, respectively. Signals at 221 and 217 nm are attributed to the n→π* transition. The region 240–300 nm is characterized by multiple signals, attributed to contribution of aromatic and thiol side chains.^[17]^ The presence of β structures, which well agrees with the computational modelling of the peptide described in the previous sections, is confirmed by the broad negative band around 215–225 nm and a significant positive band at 195 nm.^[18]^ The β structuration preferentially appears in the FYFCFYF peptide derivative with respect to the PEGylated analogue. The secondary structure characterization was furthermore executed using FTIR spectroscopy. The absorbance spectral deconvolution of the amide I region (1700‐1600 cm^−1^) for both FYFCFYF (green line) and PEG8‐FYFCYFY (blue line) at 2.0 mg/mL are reported in Figure 5c. This spectral window is specifically sensible to peptide secondary structuration.^[19]^ Deconvolution profiles for both the peptides are dominated by a principal band located at 1637 and 1640 cm^−1^ for FYFCFYF and PEG8‐FYFCFYF, respectively. In the spectrum of FYFCFYF, the association of the band at 1637 cm^−1^, with an additional signal at 1675 cm^−1^, indicates the presence of β‐sheet secondary structure with an antiparallel orientation of strands. The 1675 cm^−1^ signal may be imputed to the presence of β‐turn structure too. However, it is almost implausible that very short peptides, such as the proposed heptapeptide, can arrange into β‐turn. In addition, the primary sequence is lacking of β‐turn prone residues, such as Gly and Pro.

The inspection of FTIR results is indicative of a predominant tendency for the unPEGylated peptide to adopt a β‐sheet conformation with respect to PEG8‐FYFCFYF, thus indicating a certain impact of the PEG polymer on the supramolecular organization. The structuration in β‐sheet rich aggregates was further confirmed qualitatively using Thioflavin T (ThT) spectroscopic assays. ThT is a dye endowed with a cationic benzothiazole structure that adopts a stabilized conformation when bound to aggregates thus inducing an enhancement and a shift of the fluorescence emission peak from 445 to 482 nm.^[20]^ The fluorescence spectra of both Cys‐peptides (10 mg/mL) upon excitation at λ=450 nm in a 50 μmol/L solution of ThT are reported in Figure 5d. In both the spectra, subtracted from the self‐fluorescence of the peptide, a peak at 482 nm is clearly visible. The emission peak appears more intense for FYFCFYF. The higher ThT emission of FYFCFYF respect to PEG8‐FYFCFYF can be explained taking into account the different molar concentration of the two peptides. Indeed, both the samples have been studied at a concentration of 10 mg/mL that corresponds to 9.7 and 6.2 mmol/L for unPEGylated and PEGylated peptides, respectively.

Taking advantage from a different molecular dye‐aggregate mechanism of interaction with respect to ANS, thus avoiding some kind of dye‐aggregates and dye‐monomers interactions, the characteristic fluorescence behaviour of ThT allowed us to estimate again the CACs (Figure S11). The extrapolated values via ThT titration (1.22 ⋅ 10^−4^ mol/L, 126 μg/mL for FYFCFYF; 1.81 ⋅ 10^−5^ mol/L, 19.1 μg/mL for PEG8‐FYFCFYF) are in line with CACs obtained using ANS, thus supporting the evidence of aggregation propensity in the found concentration ranges.

All these results are in good agreement with the FTIR analysis, which highlighted a higher grade of β‐sheet organization for unPEGylated derivative with respect to PEG8‐FYFCFYF.

## Wide‐angle X‐ray scattering (WAXS)

**Figure 6:**
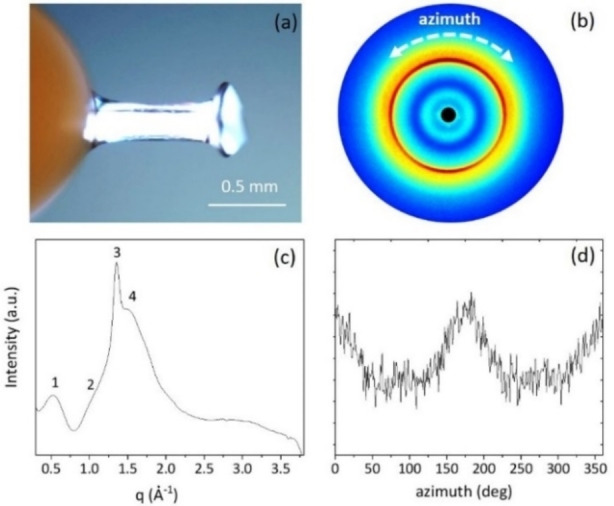
a) Image of the investigated FYFCFYF dried fiber (scale bar=0.5 mm); b) 2D WAXS data of the FYFCFYF fiber; c) radial integrated 1D WAXS profile; d) intensity distribution along the azimuth of the equatorial peak at q_1_=0.58 Å^−1^ peak.

The FYFCFYF fibril in Figure 6a, obtained by the stretch frame method, was characterized through WAXS investigation. The 2D WAXS data, reported in Figure 6b, show several continuous diffraction rings, which indicate the absence of a clear preferred orientation within the illuminated volume of the fiber. The collected 2D WAXS pattern, once centred and calibrated, and radially folded into a 1D profile (Figure 6c), reveals that the diffraction pattern has main peaks at q_1_=0.58 Å^−1^ (d_1_=10.8 Å) and q_3_=1.32 Å^−1^ (d_3_= 4.75 Å) that are in agreement with the structural models described in Figure 4, and explained as the inter‐sheet distance detected in the trajectory structures and the inter‐strand distance within each sheet, respectively. Two additional peaks were found at q_2_=1.05 Å^−1^ (d_2_=6.0 Å), and q4=1.53 Å^−1^ (d_4_=4.0 Å^−1^) too. The 1D WAXS azimuthal profile in Figure 6d indicates a slight preferred orientation mainly for the peak at q_1_=0.58 Å^−1^ that typically identifies the equatorial direction, i. e. the direction perpendicular to the fiber axis.

## Gel formation and oxidation

Interconnected fibers network can generate self‐supporting hydrogels as a consequence of additional physical cross‐linking phenomena or chemical modifications. In the specific case of peptide‐based HGs, these materials are formed by further entanglement of supramolecular fibrillary structures, which can be induced by increasing the total sample concentration, and as a consequence, the number of fibers and their mutual contacts.^[21]^ During the preparation of FYFCFYF solutions, we observed a viscosity degree that increases proportionally to the peptide concentration. Hence, we studied the capability of the two peptides to gelificate using the inverted test tube in the range of concentration between 0.25 and 2.0 wt%. As reported in Figure S12, the critical gelation concentration (CGC) for FYFCFYF can be identified in the range 0.5 wt%<CGC<1.0 wt%. On the contrary, PEG8‐FYFCFYF was not able to gelificate (data not shown). These evidences indicate a completely opposite behaviour of Cys‐containing peptides respect to the ancestor peptide (FY)3, which gelificates only in its PEGylated form.^[9]^ However, the characterization of others analogues of (FY)3 and PEG8‐(FY)3 [*eg*. (F‐Dopa)3 and (Nal‐Dopa)3 and their PEGylated versions PEG8‐(F‐Dopa)3 and PEG8‐(Nal‐Dopa)3] highlighted a diametrically opposed behavior respect to (FY)3.^[10]^ All these evidences point out the importance of the hydrophilic/hydrophobic balance in the peptide sequence in the gelification process. Moreover, it is possible that the Cys side chain plays a role in the confinement and retention of water molecules in the gel. The stability of the FYFCFYF hydrogel (macroscopically evaluated in terms of homogeneity, opacity and reproducibility) and its capability to hold water (as difference between the weight of the matrix before and after an incubation in water overnight at room temperature) were evaluated by the swelling value *q* (See Equation (1)).^[22]^ Results indicate that hydrogels at 1.0 wt% have a partial instability after the incubation in term of matric homogeneity and opacity and lower *q*=15 %, meanwhile a swelling value of 29 % and 33 % was measured for 1.5 and 2.0 wt% samples, respectively. The shelf stability was also evaluated via inverted test tube. The 1.0 wt% samples resulted less stable (20 days) respect to the samples at 1.5 wt% (up to 40 days) and to the samples at 2.0 wt% (up to 65 days), which did not report visible modification of the matrices. These evidences suggested us that the increase of concentration can improve the hydrogel stability. In addition, no syneresis was noted for 1.5 wt% and 2.0 wt% HGs, meanwhile for the 1.0 wt% sample the expulsion of water (∼15 %) occurs after five days from the preparation.

In order to further improve the hydrogel stability, we tried to generate additional chemical networks in the supramolecular material by preparing HGs in presence of ammonium bicarbonate (NH_4_HCO_3_, AmBic) at different concentrations (10.0, 6.0, 5.0, 2.0 and 1.0 mmol/L). The AmBic has been used in several peptide synthesis protocols for achieving the air oxidation of Cysteine residues with consequential formation of disulphide bonds.^[23]^ The preparation of FYFCFYF hydrogels in presence of AmBic, at different concentrations, has a different impact on both macroscopical aspect and on kinetics of HGs formation. Indeed, using AmBic solution at a concentration of 10 mmol/L, it is noted an inhomogeneous HG formation; meanwhile self‐supporting and translucent HGs were formed using a concentration of 1.0, 2.0 or 6.0 mmol/L. It was also observed a different correlation between the AmBic concentration and the gelation kinetics, with a gelation time of 35 and 45 minutes for a concentration of 2.0 and 1.0 mmol/L, respectively and an instantaneous gelation for a concentration of 6.0 mmol/L. The disulphide bond formation was experimentally checked by ESI‐MS analysis (Figure S13a), which shows the prevalent presence of dimeric species.

**Figure 7:**
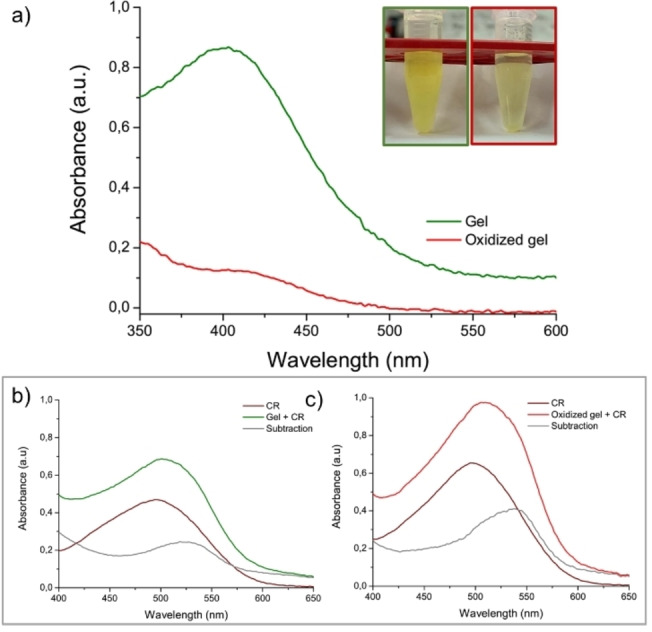
UV‐Vis spectra of oxidized and not oxidized peptide hydrogels co‐incubated with the Hellman's reagent (DTNB). Macroscopical appearance of hydrogels analyzed by UV‐Vis are reported in the insert. Absorbance spectra of CR alone or coincubated with hydrogels before (b) and after the oxidation (c). The subtraction of spectra is also reported.

The formation of an additional chemical network affects also on the CGC and swelling features. Indeed, the sample prepared in the presence of 6.0 mmol/L AmBic exhibits a moderate decrease of the critical gelation concentration (0.25 wt%<CGC<0.50 wt%) and an increase of the swelling ratio (*q* up to 34 % and 40 % for 1.5 and 2.0 wt% samples, respectively) and of the shelf stability (up to three months). The efficiency of oxidation procedure was evaluated using the well‐assessed Ellman's test.^[24]^ Ellman's reagent (5,5’‐dithio‐bis‐(2‐nitrobenzoic acid)) also known as DTNB, is a chromogenic compound used to quantify free sulfhydryl groups in solution. DTNB has an oxidizing disulphide bond that, in the presence of free thiols, undergoes reduction, releasing one molecule of 5‐thio‐2‐nitrobenzoic acid (TNB, yellow) and a mixed disulphide as products. Thus, free thiol concentration can be indirectly determined by TNB UV‐Vis absorption at 412 nm. The reaction mechanism is reported in Figure S13b. The DTNB solution added in FYFCFYF gel aliquot immediately becomes more coloured with respect to oxidized gel (see insert in Figure 7).

The β‐sheet structuration seems not to be altered by the matrix preparation via AmBic. This evidence is supported by the positive response of both HGs to Congo Red ((3,3‐([1,1’‐biphenyl]‐4,4’‐diyl)‐bis(4‐aminonaphthalene‐1‐sulfonic acid) sodium salt, CR) spectroscopic assay^[25]^ and to the FTIR analysis. UV‐Vis spectra of Cys‐containing HGs (1.5 wt%), pure or prepared using AmBic and co‐incubated with CR, are reported in Figures 7b and 7c in comparison with the spectrum of the CR alone. Both the samples exhibit a bathochromic shift of the CR maximum from 480 to ∼540 nm, as clearly visible from the spectrum subtraction, thus indicating a retention of the β‐sheet secondary structuration after Cys oxidation.

## Scanning electron microscopy (SEM) characterization

**Figure 8:**
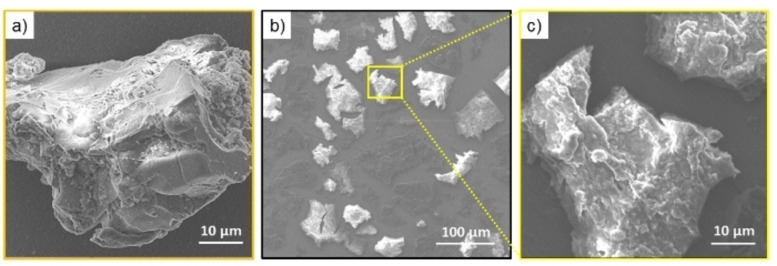
SEM microphotos of FYFCFYF xerogels before (a) and after oxydation (b,c).

Morphological characterization of hydrogels before and after oxidation was assessed by Scanning Electron Microscopy (SEM). Selected SEM microphotos of peptide xerogels are reported in Figure 8.

Contrarily to the parental (FY)3 hexapeptide^[9]^ and to the other analogues containing DOPA and Nal residues in place of Tyr and Phe ones,^[10]^ FYFCFYF hydrogel does not show a dense network of interconnected fibres, but mesoscopic fibrillary clusters that interact themselves by intermolecular forces. This kind of organization is typically observed in solid‐like colloidal gels.^[26]^ After the disulphide bridge formation an increase of clusters can be observed in the SEM microphotos, even if it can be not excluded the effect due to the increase of the ionic strength caused by AmBic.

## Rheological studies

The viscoelastic behaviour and mechanical features of FYFCFYF gels have been evaluated via rheological studies, reporting the results as G’ (Storage modulus) and G’’ (Loss modulus). For this scope, time sweeps oscillatory measurements (1.0 Hz and 0.1 % strain) were carried out on FYFCFYF gel at a concentration of 1.5 wt%; the oxidized version was prepared at the same concentration using 6 mmol/L AmBic.

Results were collected after preliminary evaluation of the optimal measurement parameters, these identified according to dynamic oscillation strain sweep (at a frequency of 1 Hz) and dynamic frequency sweep (at 0.1 % strain) for both the tested gels (Figure S12).

**Figure 9:**
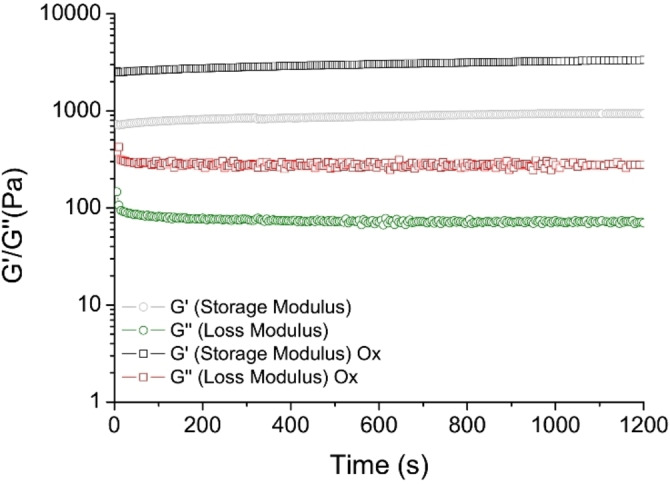
Time sweep (20 minutes) for FYFCFYF gel and for FYFCFYF oxidized one. Rheological analysis is reported in terms of G’ (Storage modulus) and G’’ (Loss modulus).

The linear viscoelastic region (LVE region), indicating the range of strain in which the rheological tests can be carried out without destroying the structure of the sample, was identified in the 0.03‐3.0 % range of the strain sweep diagram for both hydrogelated matrices. G’ and G’’ time sweep profiles in Figure 9, acquired for 20 minutes at a frequency of 1 Hz and at 0.1 % strain, evidence values of G’ higher than G’’, thus analytically confirming the gel state of the samples.^[27]^ FYFCFYF gel possesses a couple of value of G’=970 Pa and G’’=71 (tan δ=13.6). These values point out that the Cys‐containing peptide is able to produce a matrix ten time more rigid if compared with the ancestor hydrogel PEG8‐(FY)3 (G’∼100 Pa).

After AmBic oxidation, G’ and G’’ moduli values increase to 3360 Pa and 273 Pa, respectively (tan δ=12.3). This increase is symptomatic of an efficient improvement of the mechanical rigidity. The enhancement of stiffness is also detectable by an increase of the breakage point of strain (from 22 % to 40 %) and of frequency (from 25 Hz to 50 Hz) (see Figure S14).

This result points out the capability of disulphide bonds to enlarge the network of interactions, thus generating an accentuated viscoelastic response of the material.

## Conclusions

In virtue of their high biocompatibility, biodegradability and to their adjustable mechanical properties, peptide‐based HGs have been proposed for a myriad of applications in different biomedical^[28]^ and biotechnological areas.^[29]^

Due to their similar structure with native extracellular matrix (ECM), as well as their mechanics similar to those of many biological tissues, HGs have emerged as promising scaffolds in tissue regeneration^[30]^ and bioink.^[31]^ Moreover, the efficiency to answer to external stimuli (such as variation of pH, temperature and salt content) and the target ability of some peptide sequences make peptide‐based HGs suitable carriers for the delivery of drugs and/or diagnostic agents.^[32]^ Furthermore, due to their capability to rapidly undergo to sol‐gel transition under the high shear rate; PHGs have been also proposed as injectable materials.^[33]^ In this scenario, the research has been focused in the last years in the design and synthesis of novel peptide building blocks able to generate hydrogels. The capability of completely aromatic peptides [(F‐Dopa)3, (Nal‐Y)3 and (Nal‐Dopa)3] and of their PEGylated derivatives [PEG8‐(FY)3 and PEG8‐(Nal‐Y)3] to self‐assemble into self‐supporting hydrogels has been recently demonstrated.[^9^, ^10^] MD simulations studies suggested that the aggregation and the gelification of these peptides are due to the presence of two interfaces: a wet and a dry one. Since the use of cross‐linking of peptides and proteins in amyloid‐like nanostructures via disulphide bond formation is progressively becoming a powerful tool for modulating their structural and functional properties,^[34]^ we here evaluated the impact of such a modification on (FY)3‐based assemblies. Our investigations demonstrate that the introduction of a cross‐linkable cysteine residue in the wet interface of the peptide (FY)3 does not hamper the gelification process. Moreover, the cross‐linking of cysteine residues generates a more rigid hydrogel, potentially useful for bone tissue regeneration. Moreover, the presence of a Cys residue in the middle of the peptide sequence could be employed for derivatization of the hydrogel with targeting moieties or with biologically relevant molecules such as drugs and/or diagnostic agents.

## Experimental Section

**Materials**: Protected N^α^‐Fmoc‐amino acids (Fmoc‐Cys(Trt)‐OH, Fmoc‐Phe‐OH and Fmoc‐Tyr(OtBu)‐OH), coupling reagents and Rink amide MBHA (4‐methylbenzhydrylamine) resin were purchased from Calbiochem‐Novabiochem (Laufelfingen, Switzerland). The monodisperse Fmoc‐8‐amino‐3,6‐dioxaoctanoic acid, [Fmoc‐AdOO‐OH, PEG2] was purchased from Neosystem (Strasbourg, France). All other chemical reagents, 1,1,1,3,3,3‐hexafluoropropan‐2‐ol (HFIP), 8‐anilino‐1‐naphthalene sulfonic acid ammonium salt (ANS), thioflavin T (ThT) and 5,5’‐dithio‐bis‐(2‐nitrobenzoic acid, DTNB), are commercially available on Merck (Milan, Italy), Fluka (Bucks, Switzerland) or LabScan (Stillorgan, Dublin, Ireland). All of them were used as received unless otherwise stated. Purifications were carried out on a LC8 Shimadzu HPLC system (Shimadzu Corporation, Kyoto, Japan) equipped with a UV lambda‐Max Model 481detector using Phenomenex (Torrance, CA) C18 column. Elution solvents are H_2_O/0.1 % TFA (A) and CH_3_CN/0.1 % TFA (B), from 20 % to 70 % over 30 minutes at 20 mL/min flow rate. Purity of products was assessed using analytical HPLC (Agilent), column: C18‐Phenomenex eluted with an H_2_O/0.1 % TFA (A) and CH_3_CN/0.1 % TFA (B) from 20 % to 80 % over 20 minutes at 1 mL/min flow rate. Identity of peptides was checked by MS analyses on LTQ XL™ linear ion trap mass spectrometer LTQ‐XL with an HESI sorgent (Finnigan Surveyor MSQ single quadrupole electrospray ionization (Finnigan/Thermo Electron Corporation San Jose, CA).

## Molecular modeling and dynamics

**Systems and notations**: Three‐dimensional models of FYFCFYF aggregates were generated following the procedure we previously reported.^[11]^ A single sheet model of FYFCFYF was generated using as template the structure of the hexapeptide fragment KLVFFA of the amyloid‐beta peptide II (Protein Data Bank entry 3OW9).^[35]^ Starting from this structure, we generated a model composed of a single fifty‐stranded β‐sheet. A steric zipper model was then produced through the association of a pair of these sheets using the organization of the KLVFFA peptide in the crystalline state (FYFCFYF_ST50_SH2). Taking into account the hydrophobicity/hydrophilicity of Tyr and Phe residues, the two‐sheet model was built by locating the Phe side chains at the dry interface leaving Tyr and Cys residues solvent exposed. A more complex system was generated by considering four ten‐stranded β‐sheets (FYFCFYF_ST10_SH4). As shown in Figure S1, this assembly is endowed with two different steric zipper interfaces: two hydrophobic ones composed of Phe side chains and a hydrophilic one made of Tyr/Cys residues. In addition, in this model two surfaces made by Tyr/Cys residues are solvent exposed. Finally, a variant of FYFCFYF_ST10_SH4 in which the Cys residues in the hydrophilic interface form Cys‐Cys disulfide bonds was also characterized (FYFCFYF_ST10_SH4 SS).

**Molecular dynamics protocol**: The GROMACS software^[36]^ was used to perform MD simulations on the models we generated (FYFCFYF_ST50_SH2, FYFCFYF_ST10_SH4 and FYFCFYF_ST10_SH4 SS). Amber03 and TIP3P were used as force field and water model, respectively. The systems were solvated with water molecules in triclinic boxes. Cl^−^ counterions were added to balance charges. MD parameters of the simulations (box dimensions, number of water molecules and ions) are reported in Table S1. Electrostatic interactions were computed by means of the particle‐mesh Ewald (PME) method with a grid spacing of 1.2 Å and a relative tolerance of 10^−6^. A 10 Å cut‐off was applied for the Lennard‐Jones (LJ) interactions. The LINCS algorithm was used for constraining bond lengths. The systems were initially energy minimized using steepest descent (50,000 steps) and then equilibrated in two phases. In the first step, systems were heated to 300 K temperature for 500 ps (NVT). Then, the pressure was equilibrated at the value of 1 atm for 500 ps (NpT). The Velocity Rescaling and Parrinello‐Rahman algorithms were used to control temperature and pressure, respectively. The MD production runs were carried out at constant temperature (300 K) and pressure (1 atm) with a time step of 2 fs. The analysis of trajectory structures was performed by using the VMD program^[37]^ and GROMACS tools. The achievement of an adequate convergence in the production runs was checked by calculating the root mean square inner product (RMSIP) values between the two halves of the equilibrated trajectories (Table S1).

**Peptide synthesis**: Peptides FYFCFYF and PEG8‐FYFCFYF were synthesized according to the solid phase peptide synthesis (SPPS) procedure previously optimized and reported.^[9]^ Briefly, Rink amide MBHA resin (substitution 0.73 mmol/g, 0.25 mmol) was allowed to swell in N, N‐dimethylformamide (DMF) for 35 minutes. Then, peptide sequences were progressively elongated by coupling the Fmoc‐protected amino acids and the Fmoc‐monodisperse oxothylene spacer twice in DMF. Each coupling was performed using active ester strategy and using a reaction time of 45 min in presence of HOBt (Hydroxybenzotriazole), HBTU ((2‐(1H‐benzotriazol‐1‐yl)‐1,1,3,3‐tetramethyluronium hexafluorophosphate) and DIPEA (N,N‐Diisopropylethylamine or Hünig's base reagent) as coupling activating agents. Peptides were cleaved from the resin by treatment with a TFA (trifluoroacetic acid) solution containing TIS (triisopropylsilane), EDT (1,2‐Ethanedithiol) and water at 92.5 %, 2.5 %, 2.5 % and 2.5 % v/v/v/v at room temperature for 3 h. Crude products were precipitated with ice‐cold ethyl ether, dissolved in H_2_O/CH_3_CN and lyophilized for three times. After lyophilization, both the peptides were purified by reverse phase high pressure liquid chromatography (RP‐HPLC). Chemical identity of the pure products was verified and assessed by ESI mass spectrometry and analytical RP‐HPLC chromatography.

*FYFCFYF characterization*: t_R_=13.65 min, MS (ESI^+^): m/z 1035.2 *calcd*. for C_57_H_62_N_8_O_9_S: [M+Na^+^] =1058.1.

*PEG8‐FYFCFYF characterization*: t_R_=12.90 min, MS (ESI^+^): m/z 1615.8 *calcd*. for C_81_H_106_ N_12_O_21_S: [M+Na^+^]=1638.8; [M+Na^+^]/2=820.4.

**Preparation of aqueous peptide solutions**: Sample solutions of FYFCFYF were prepared using a HFIP as co‐solvent. A 100 mg/mL solution was prepared and then diluted in water. The organic solvent was removed using N_2_ flow. Peptide solutions of PEG8‐FYFCFYF were prepared by simply dissolving the lyophilized powders in water at the desired concentration, and sonicating them for 30 minutes. The concentration of the solutions was spectroscopically determined by absorbance on UV‐Vis Thermo Fisher Scientific Inc (Wilmington, Delaware USA) Nanodrop 2000c spectrophotometer equipped with a 1.0 cm quartz cuvette (Hellma) using a molar absorptivity (ϵ) of 3210 M^−1^ cm^−1^ for both the peptides.

**Dynamic Light Scattering (DLS) measurements**: The hydrodynamic diameter of peptide aggregates were measured by DLS, carried out on a Zetasizer Nano ZS instrument (Malvern Instruments, Westborough, MA) employing a 173 backscatter detector. Other instrumental initial settings, optimized automatically by the instrument, were: measurement position point=4.54 mm; attenuator=7; temperature=25 °C; cell=1.0 mL disposable sizing cuvette. DLS measurements were carried out in triplicate on aqueous samples at 2.0 mg/mL after a centrifugation step operated at room temperature at 13000 rpm for 4 minutes.

**Fluorescence studies**: The determination of the critical aggregate concentration (CAC) for the peptides was assessed by a fluorescence spectroscopy method, in which the two fluorescent dyes ANS (8‐anilino‐1‐naphthalene sulfonic acid ammonium salt) and Thioflavin T are titrated with the peptide solution. In details, the experiment was carried out by adding small aliquots of peptide derivatives in 200 μL of 20 μmol/L ANS water solution or in 200 μL of 50 μmol/L ThT water solution The emission spectra were recorded at room temperature with a spectrofluorophotometer Jasco (Model FP‐750) and the sample was located in a quartz cell with 1.0 cm path length. The others setting are: excitation and emission bandwidths=5 nm; recording speed=125 nm/min and automatic selection of the time constant, and λ_ex_=350 nm (for ANS) and λ_ex_=450 (for ThT). Spectra were then corrected for the blank and adjusted for dilution. Fluorescence emission spectra of FYFCFYF and PEG8‐FYFCFYF at several peptide concentrations (0.05, 0.1, 0.25, 0.5, 1.0, 2.0, 4.0 and 10 mg/mL) were also recorded by exciting samples both at λ_ex_=257 and λ_ex_=276 nm.

**Circular Dichroism**: Far‐UV CD spectra of FYFCFYF and PEG8‐FYFCFYF peptides were collected with a Jasco J‐810 spectropolarimeter equipped with a NesLab RTE111 thermal controller unit using a 0.1 mm quartz cell at 25 °C. The spectra were recorded from 300 to 190 nm on water solutions of samples at 1.0 mg/mL. Other experimental settings were: scan speed=10 nm/min, sensitivity=50 mdeg, time constant=16 s, bandwidth=1 nm. Each spectrum was obtained by averaging three scans and corrected for the blank. Here Θ represents the mean residue ellipticity (MRE), i. e. the ellipticity per mole of peptide divided by the number of amide bonds in the peptide sequences.

**Fourier Transform Infrared spectroscopy (FTIR)**: FTIR spectra of both the peptides solubilized at a concentration of 2.0 mg/mL were collected on a Jasco FT/IR 4100 spectrometer (Easton, MD) in an attenuated total reflection (ATR) mode and using a Ge single‐crystal at a resolution of 4 cm^−1^. Each spectrum was obtained by recording a total of 120 scans with a rate of 2 mm/s against a KBr background. After collection in transmission mode, spectra were converted directly in their deconvolution in emission.

**Thioflavin T (ThT) spectroscopic assay**: Cys‐containing peptide conjugates were studied using Thioflavin T (ThT). Fluorescence spectra of an aqueous solution of ThT (50 μM) before and after the addition of peptide derivatives (10.0 mg/mL) was recorded at 25 °C after the peptide addition into the cuvette. Samples were excited at 450 nm and fluorescence emission spectra were recorded between 460 and 600 nm. Spectra are reported after subtraction of ThT and peptide aggregates alone.

**Wide Angle X‐Ray Scattering (WAXS)**: FYFCFYF fiber (Figure 6a) was prepared from stalks using the ‘‘stretch‐frame’’ method.^[38]^ 2D WAXS data were collected at the X‐ray MicroImaging Laboratory (XMI−L@b), equipped with a Fr−E+ SuperBright rotating anode table‐top microsource (Cu K_α_, λ= 0.15405 nm, 2475 W), a multilayer focusing optics (Confocal Max‐Flux; CMF 15–105) and a three‐pinholes camera (Rigaku SMAX‐3000). An image plate (IP) Raxia detector with 100 μm pixel size and off‐line reader was placed at around 10 cm from the sample to acquire the data. Once acquired, the 2D WAXS data were centered, calibrated by means of the Si NIST standard reference material (SRM 640b) and folded into 1D WAXS radial profiles.^[39]^

**FYFCFYF hydrogel preparations**: A volume of 400 μL of peptide HG was prepared via dilution in water at different final concentration of a HFIP stock solutions (200, 100 or 50 mg/mL). The HG is immediately formed after the addition of water to the organic solvent. The oxidized HG was prepared by hydrating the organic solvent with NH_4_HCO_3_ ammonium bicarbonate (AmBic) solutions (10.0, 6.0, 5.0, 2.0 and 1.0 mmol/L).^[23]^ In all the preparations, HFIP was gently removed using N_2_ flow, avoiding any physical stress.

**Swelling test**: The swelling ratio of hydrogels (*q*) was evaluated by adding 1.4 mL of doubly distilled water to each sample of 400 mL at different concentrations. After an overnight incubation at room temperature, swollen matrices were weighed (W_s_) immediately after the removal of aqueous medium. Then, the samples were freeze‐dried and weighed again (W_d_). The swelling behavior was expressed, according to Equation (1), as the swelling ratio *q* that corresponds to the ratio between the weight of the swollen sample (W_s_) and the weight of the freeze‐dried hydrogel (W_d_):

(1) $$q=\;\frac{\left(Ws-Wd\right)}{Wd}\%$$

**Ellman's Test**: The test was conducted using the chromogenic reagent 5,5’‐dithiobis‐(2‐nitrobenzoic) acid (DTNB) to determine the free sulfhydryl groups in gels, in normal or oxidizing conditions. The DTNB was prepared at 2.0 mmol/L in a 50 mmol/L sodium acetate solution (stock A). A solution of Tris ⋅ HCl at a concentration of 1.0 mol/L was adjusted to pH=8.0 (stock B). Reagent solution (stock C) was obtained by mixing 50 μL of stock A, 100 μL of stock B and 840 μL of H_2_O. 20 μL of gels were added to 980 μL of stock C. Samples were vortexed, properly diluted and incubated at room temperature for 10 minutes. Then, free thiols were indirectly quantified via UV‐Vis measurements at 412 nm (ϵ=13600 cm^−1^ ⋅ mol^−1^ ⋅ L), acquiring the spectrum of the formed 5‐thio‐2‐nitrobenzoic ion (TNB) yellow solutions.

**Scanning Electron Microscopy (SEM)**: Morphological analysis of xerogels was carried out by field emission scanning electron microscope (PhenomXL, Alfatest). 10 μL of peptide hydrogel were drop‐casted on an aluminium stub and air‐dried. A thin coat of gold and palladium was sputtered at a current of 25 mA for 75 sec. The sputter coated samples were then introduced into the specimen chamber and the images were acquired at an accelerating voltage of 10 kV, spot 3, through the Secondary Electron Detector (SED).

**Rheological characterization**: The rheological properties of the gels were evaluated using a rotational controlled stress rheometer (Malvern Kinexus) using a 15 mm flat‐plate geometry (PU20:PL61). Freshly prepared hydrogel sample (400 μL) at a concentration of 1.5 wt% was used. Each experiment was performed at 25 °C using a humidity chamber and a gap of 1 mm. Preliminary dynamic rheological tests were carried out in order to identify the regime of linear viscoelasticity. The viscous elastic region was determined by oscillatory frequency (0.1–100 Hz) and strain sweep (0.01–100 %). Then a time‐sweep oscillatory evaluation test (using a constant 0.1 % strain and 1 Hz frequency) was performed for 20 minutes. Results are reported in Pascal (Pa) as shear Storage or elastic modulus (G’) and the Shear loss or viscous modulus (G”).

## Conflict of interest

The authors declare no conflict of interest.

## Supporting information

As a service to our authors and readers, this journal provides supporting information supplied by the authors. Such materials are peer reviewed and may be re‐organized for online delivery, but are not copy‐edited or typeset. Technical support issues arising from supporting information (other than missing files) should be addressed to the authors.

Supporting Information
