# Supplementary material for: The Introduction of a Cysteine Residue Modulates The Mechanical Properties of Aromatic‐Based Solid Aggregates and Self‐Supporting Hydrogels
Source: Chemistry. 2021 Oct 1;27(60):14886–98. doi: 10.1002/chem.202102007 (PMC8596998; doi:10.1002/chem.202102007)
Supplement: Supplementary file 1 — Supporting Information [file CHEM-27-14886-s001.pdf]

# Chemistry–A European Journal

Supporting Information


## The Introduction of a Cysteine Residue Modulates The Mechanical Properties of Aromatic-Based Solid Aggregates and Self-Supporting Hydrogels


Carlo Diaferia, Elisabetta Rosa, Nicole Balasco, Teresa Sibillano, Giancarlo Morelli, Cinzia Giannini, Luigi Vitagliano, and Antonella Accardo*

## Author Contributions

C.D. Conceptualization:Lead; Data curation:Lead; Investigation:Lead; Methodology:Equal; Software:Lead; Supervision:Supporting; Validation:Lead; Writing – original draft:Equal
E.R. Data curation:Lead; Investigation:Equal; Methodology:Equal; Writing – review & editing:Supporting
N.B. Data curation:Equal; Formal analysis:Lead; Investigation:Equal; Methodology:Lead; Software:Lead; Validation:Supporting
T.S. Data curation:Equal; Formal analysis:Equal; Investigation:Equal; Methodology:Equal; Writing – original draft:Equal
G.M. Funding acquisition:Lead; Resources:Equal; Supervision:Equal; Writing – original draft:Lead; Writing – review & editing:Lead
C.G. Data curation:Lead; Investigation:Equal; Methodology:Equal; Resources:Lead; Validation:Equal; Writing – original draft:Equal; Writing – review & editing:Equal
L.V. Conceptualization:Lead; Data curation:Lead; Formal analysis:Equal; Funding acquisition:Supporting; Investigation:Equal; Methodology:Lead; Software:Supporting; Validation:Equal; Writing – original draft:Equal; Writing – review & editing:Lead
A.A. Conceptualization:Lead; Data curation:Lead; Formal analysis:Lead; Funding acquisition:Lead; Investigation:Lead; Supervision:Lead; Writing – original draft:Lead; Writing – review & editing:Lead

**Table S1.** Overall parameters of the MD simulations.

| System | Box dimension ($nm^3$) | Water molecules | Time (ns) | RMSIP |
|---|---|---|---|---|
| FYFCFYF_ST50_SH2 | 5.074x26.055x4.399 | 14764 | 500 | 0.87* |
| FYFCFYF_ST50_SH3 | 5.542x26.123x5.495 | 19442 | 1000 | 0.84[#] |
| FYFCFYF_ST10_SH3_SS | 5.249x7.012x5.171 | 4942 | 1000 | 0.73[#] |

*this value has been calculated between the two halves (250-375 ns and 375-500 ns) of the trajectory
[#]this value has been calculated between the two halves (500-750 ns and 750-1000 ns) of the trajectory

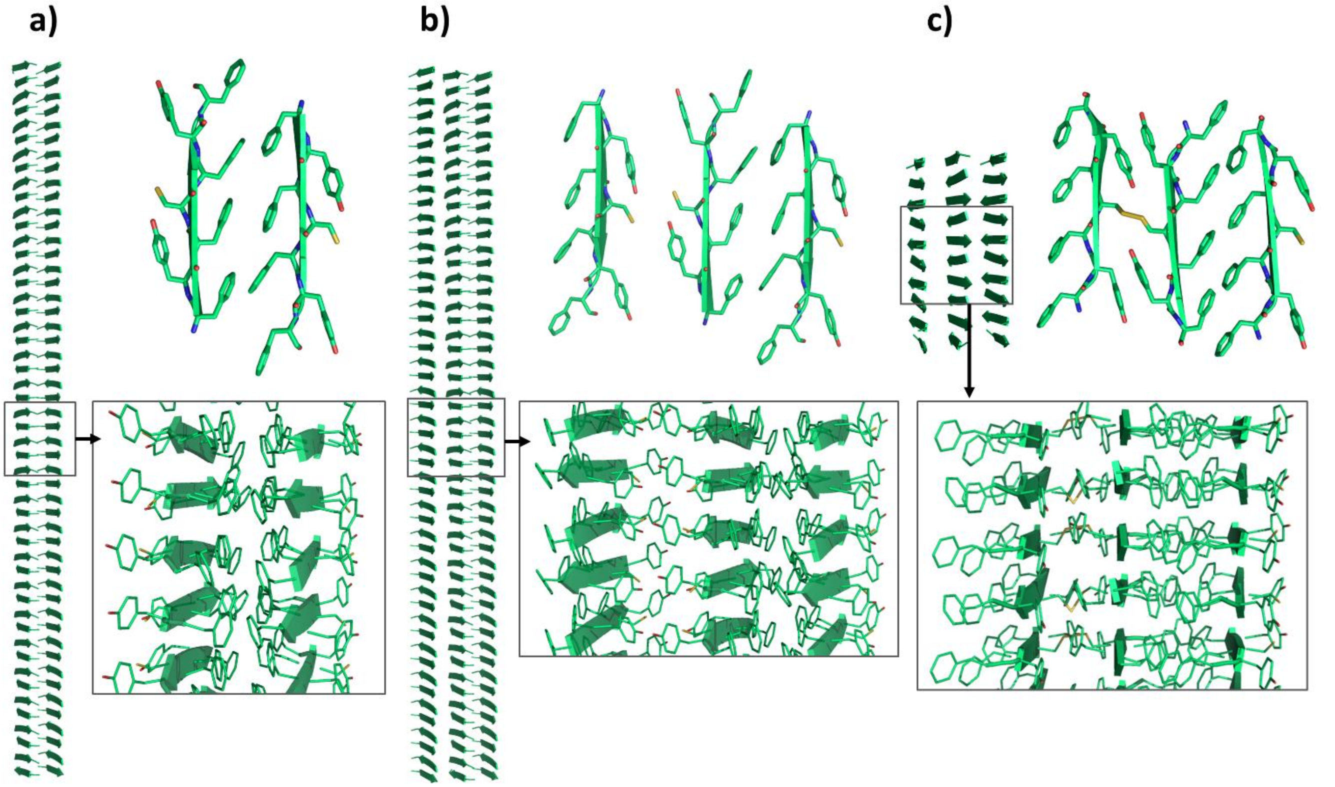


**Figure S1:** Three-dimensional representations of the starting models used in the molecular dynamics studies: FYFCFYF_ST50_SH2 (a), FYFCFYF_ST50_SH3 (b), and FYFCFYF_ST10_SH3_SS (c).

a) b)

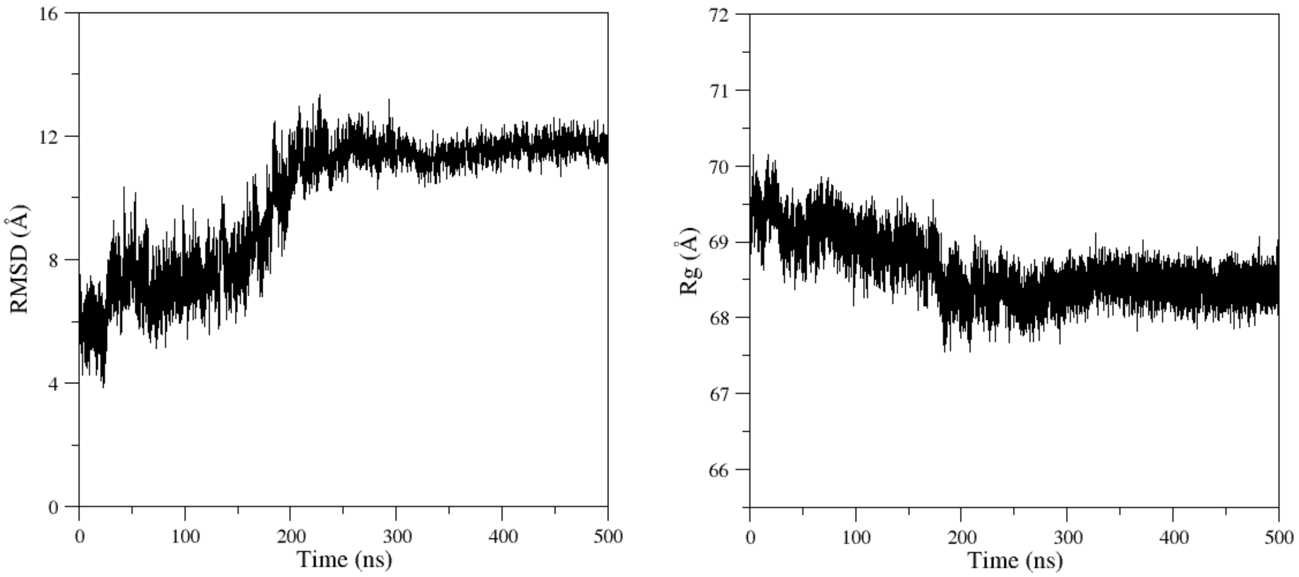


**Figure S2.** Time evolution of several structural parameters in the MD simulation performed starting from the flat model of FYFCFYF_ST50_SH2: a) RMSD values computed on the $C^{\alpha}$ atoms of trajectory structures against the starting model and b) gyration radius.

**a)**

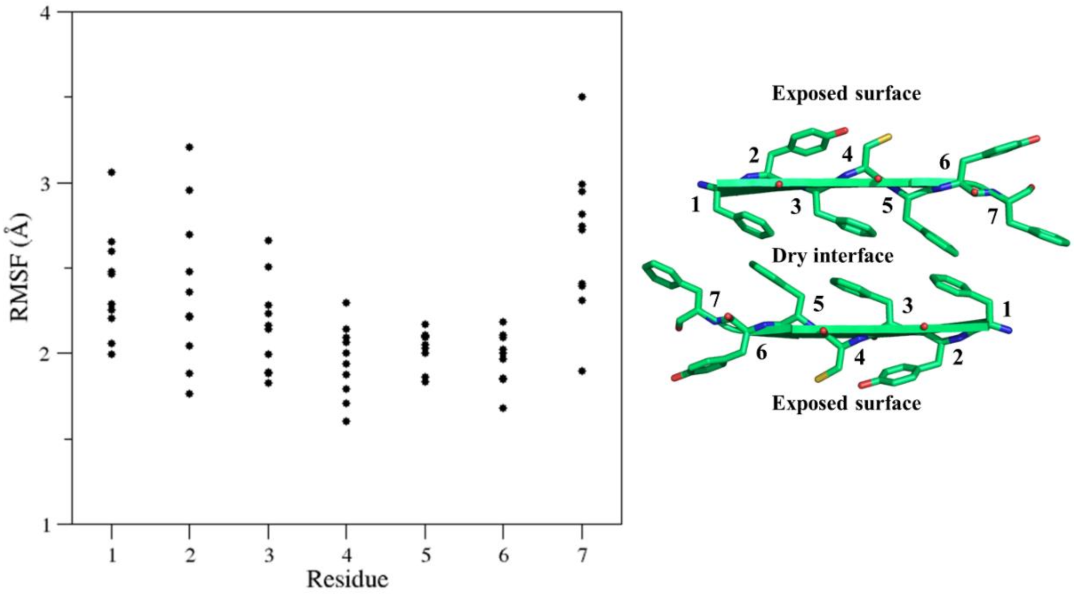


**b)**

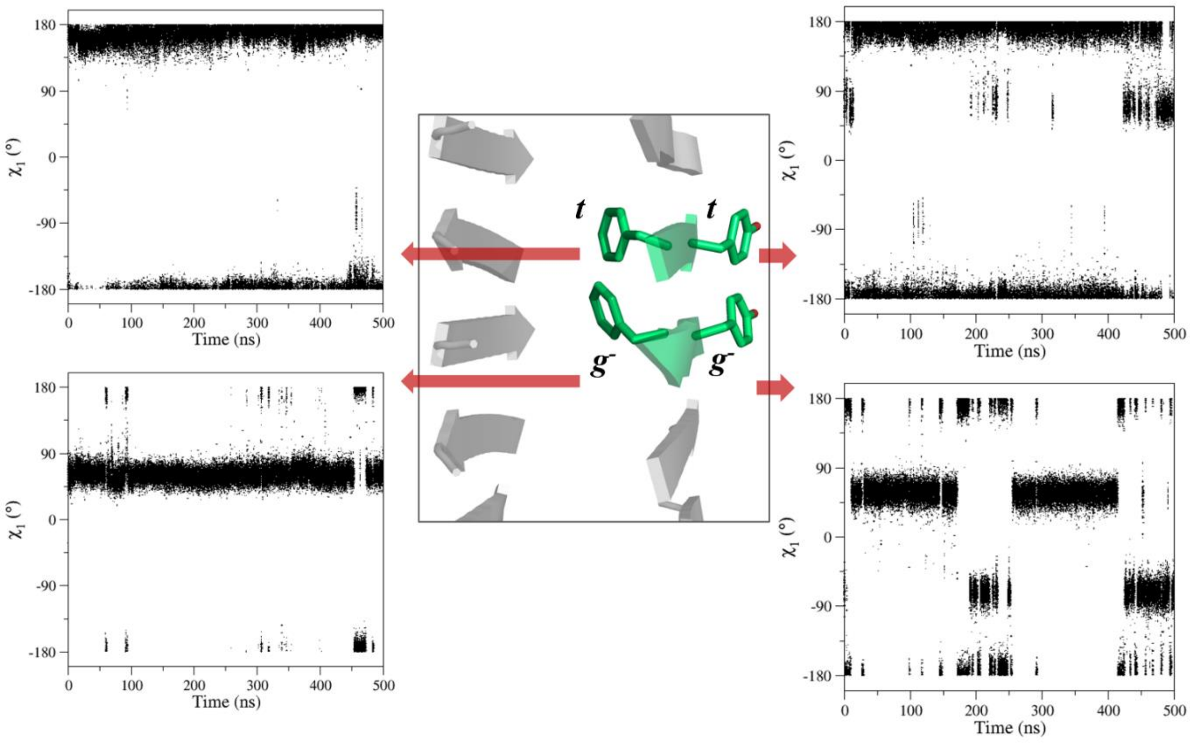


**Figure S3.** a) RMSF values computed on the side chains of the FYFCFYF_ST50_SH2 model in the equilibrated region of the trajectory (250–500 ns). For clarity, only the values of the central ten β-strands (five strands *per* sheet) are reported. b) Representative examples of the time evolution of the χ1 dihedral angle of Phe/Tyr side chains.

a) b)

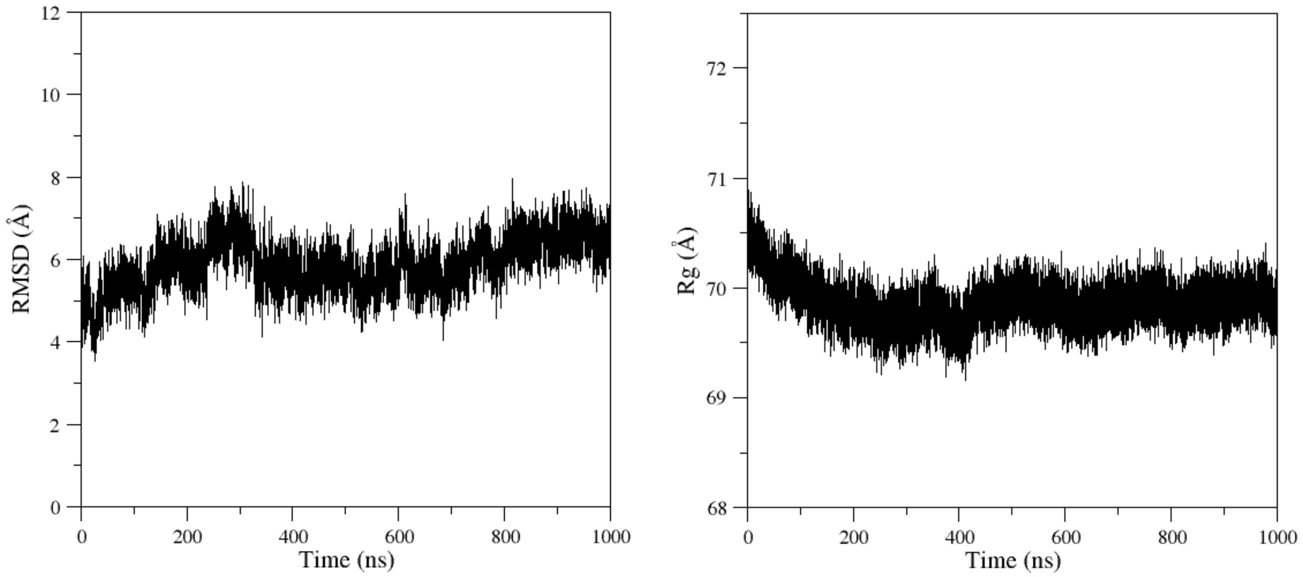


**Figure S4.** Time evolution of several structural parameters in the MD simulation performed starting from the flat model of FYFCFYF_ST50_SH3: a) RMSD values computed on the $C^{\alpha}$ atoms of trajectory structures against the starting model and b) gyration radius.

**a)**

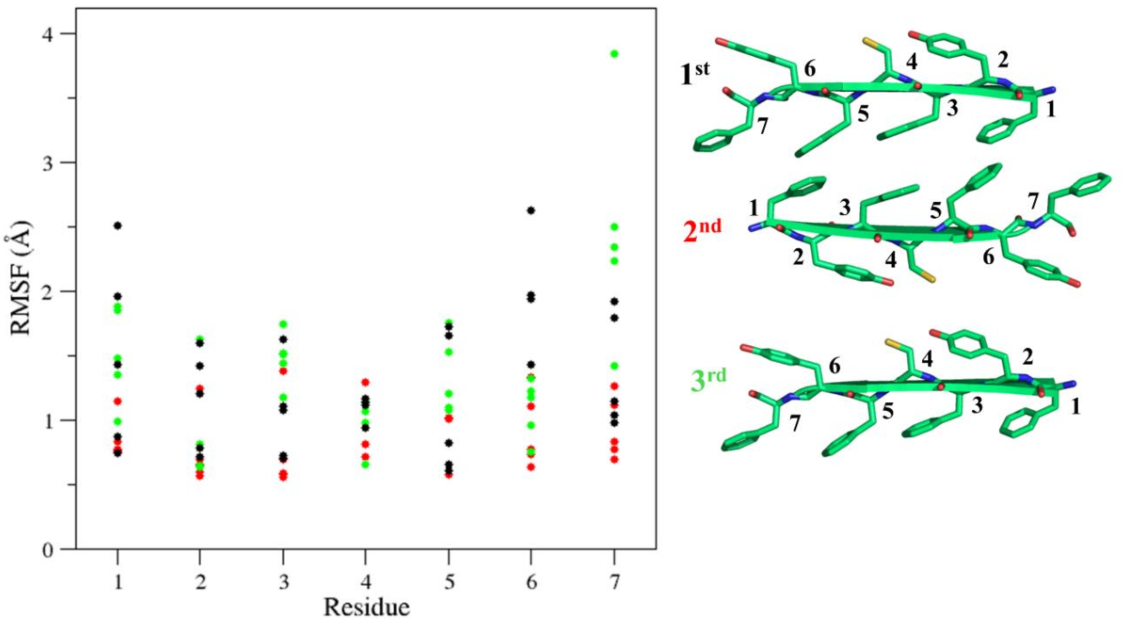


**b)**

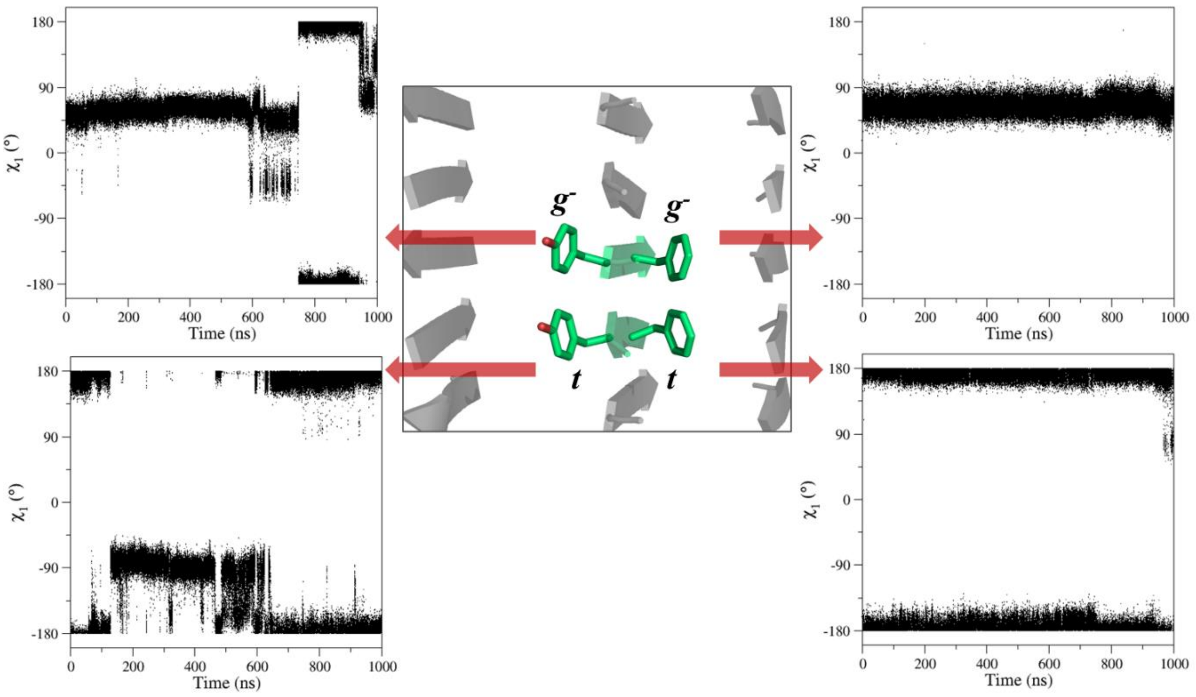


**Figure S5.** a) RMSF values computed on the side chains of the FYFCFYF_ST50_SH3 model in the equilibrated region of the trajectory (500–1000 ns). For clarity, only the values of the central fifteen β-strands (five strands *per* sheet) are reported. b) Representative examples of the time evolution of the χ1 dihedral angle of Phe/Tyr side chains of the central β-sheet.

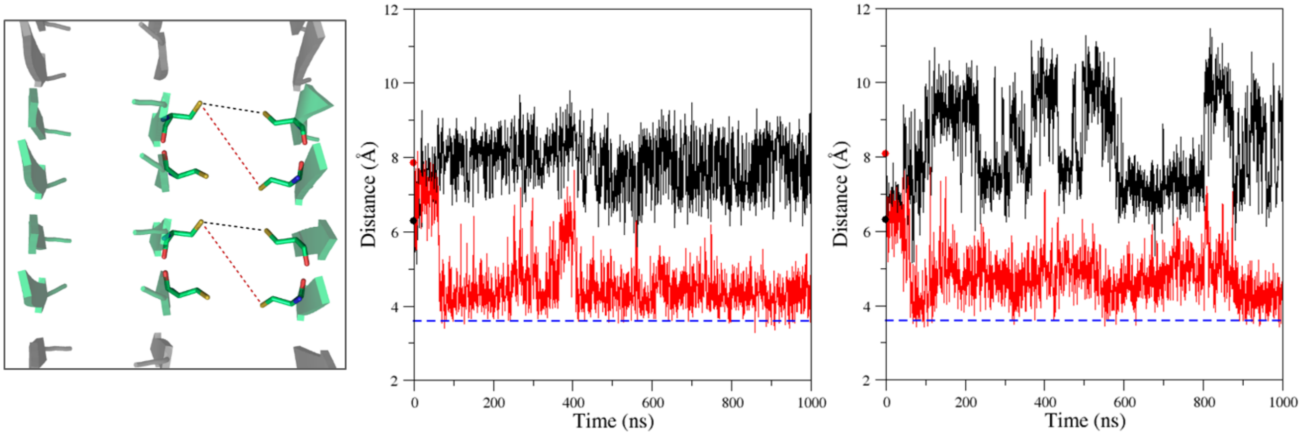


**Figure S6.** Representative examples of the time evolution of the S-S distance between the side chains of the Cys residues located in facing sheets in the MD simulation of FYFCFYF_ST50_SH3. The dashed blue line represents the theoretical interatomic distance (3.6 Å) between sulfur atoms based on their van der Waals radius (~ 1.8 Å). The color of the line (black or red) corresponds to the color that designates the corresponding distance as a dashed line in the left panel. The black/red dot reported on the vertical axis represent the value of the distance at t=0 ns.

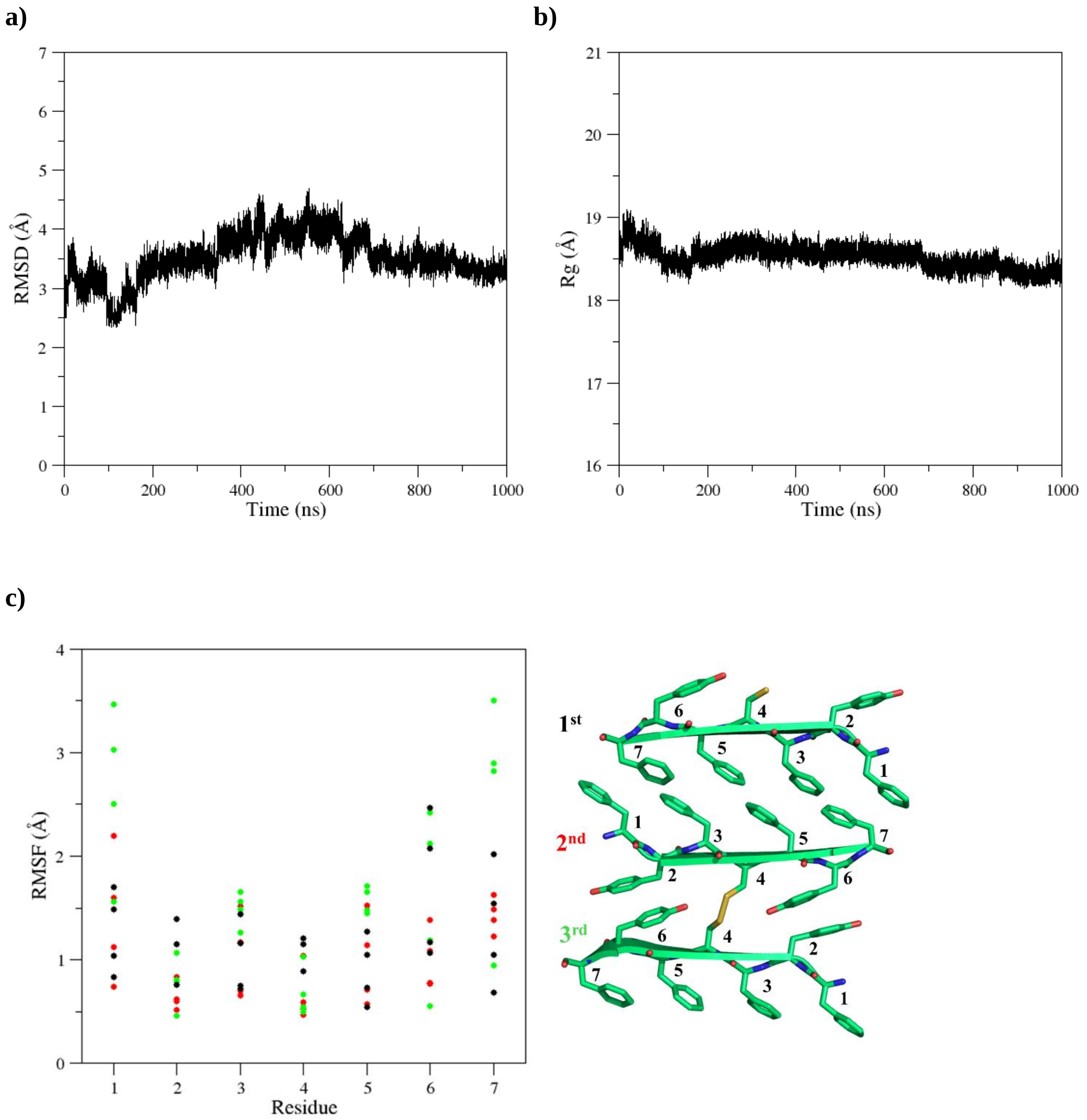


**Figure S7.** Time evolution of several structural parameters in the MD simulation performed starting from the flat model of FYFCFYF_ST10_SH3: a) RMSD values computed on the $C^{\alpha}$ atoms of trajectory structures against the starting model and b) gyration radius. c) RMSF values computed on the side chains of the FYFCFYF_ST10_SH3_SS model in the equilibrated region of the trajectory (700–1000 ns). For clarity, only the values of the central twelve β-strands (four strands *per* sheet) are reported.

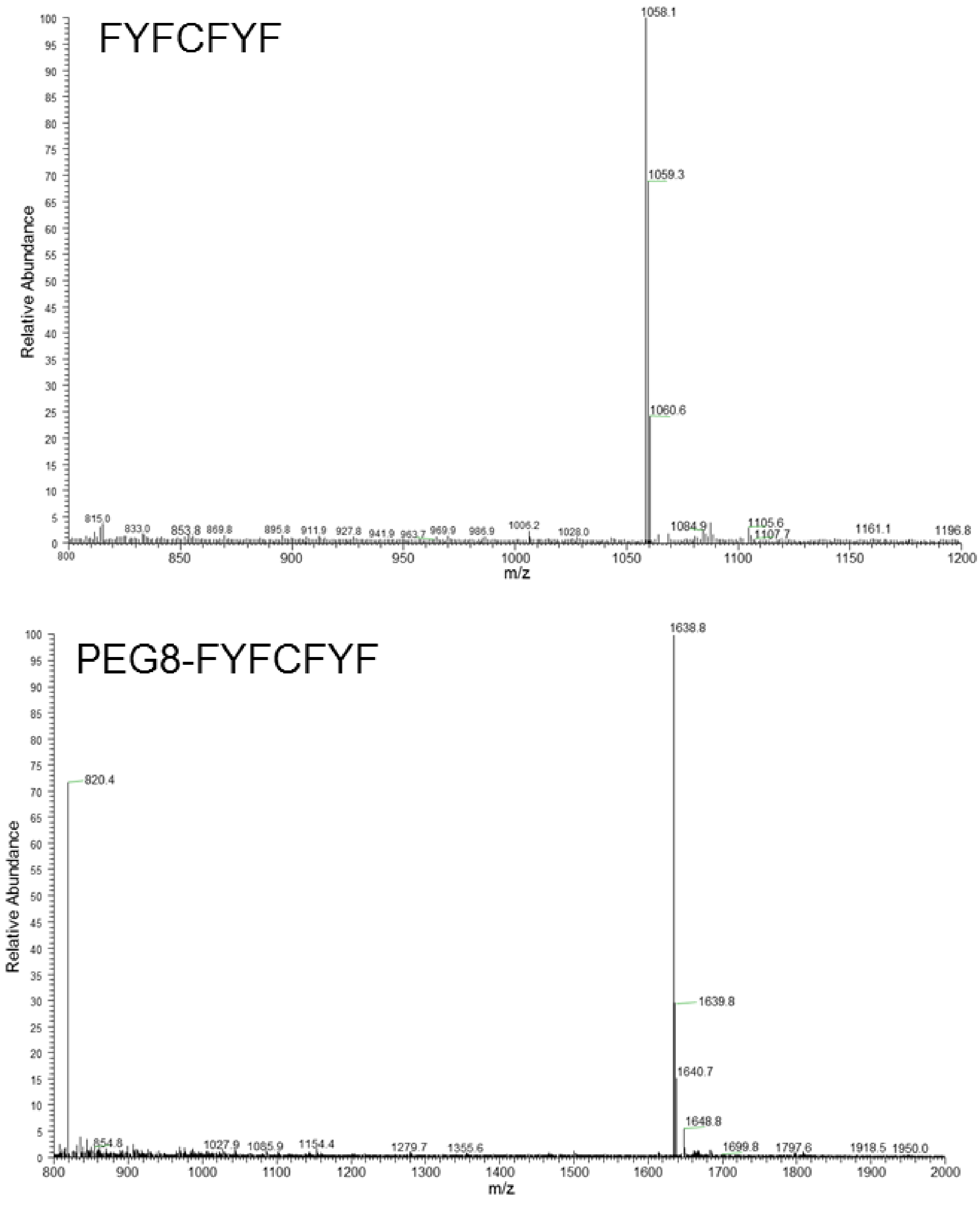


**Figure S8.** ESI-MS analysis for both Cys-containing peptides.

a)

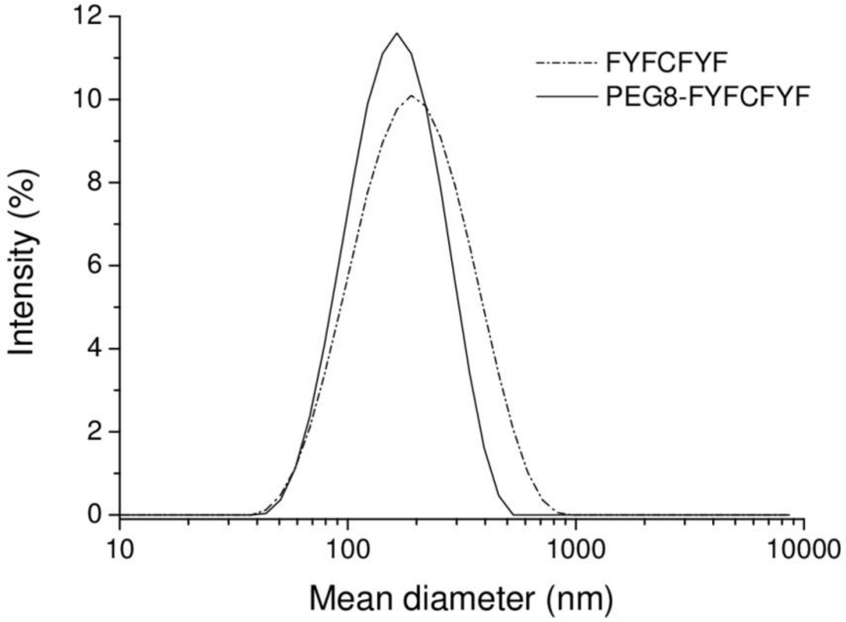


b)

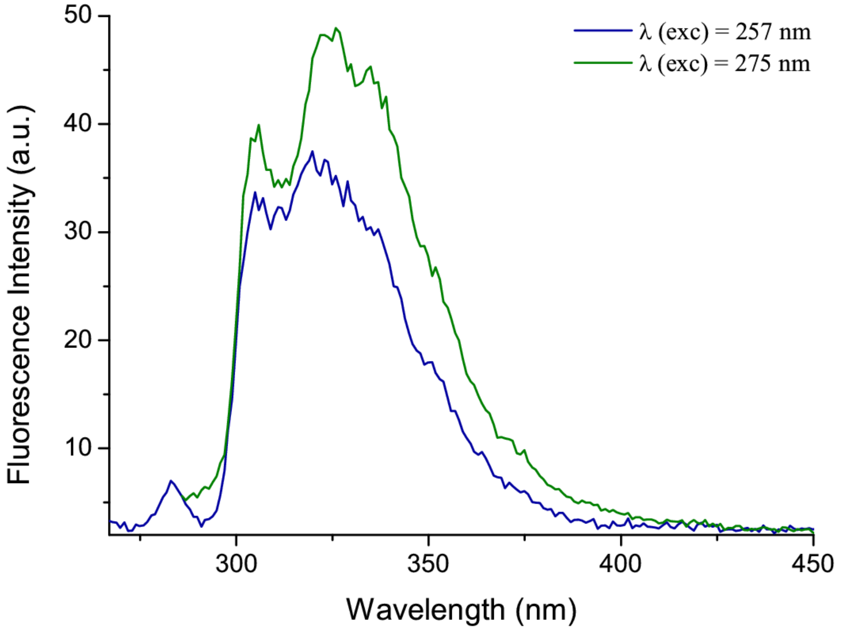


**Figure S9:** a) DLS intensity profiles of peptide aggregates recorded at 2.0 mg/mL. b) Emission spectra for PEG8-FYFCFYF peptide solution at 0.005 mg/mL. Excitation wavelength corresponding to λ=257 nm and λ=275.

a)

b)

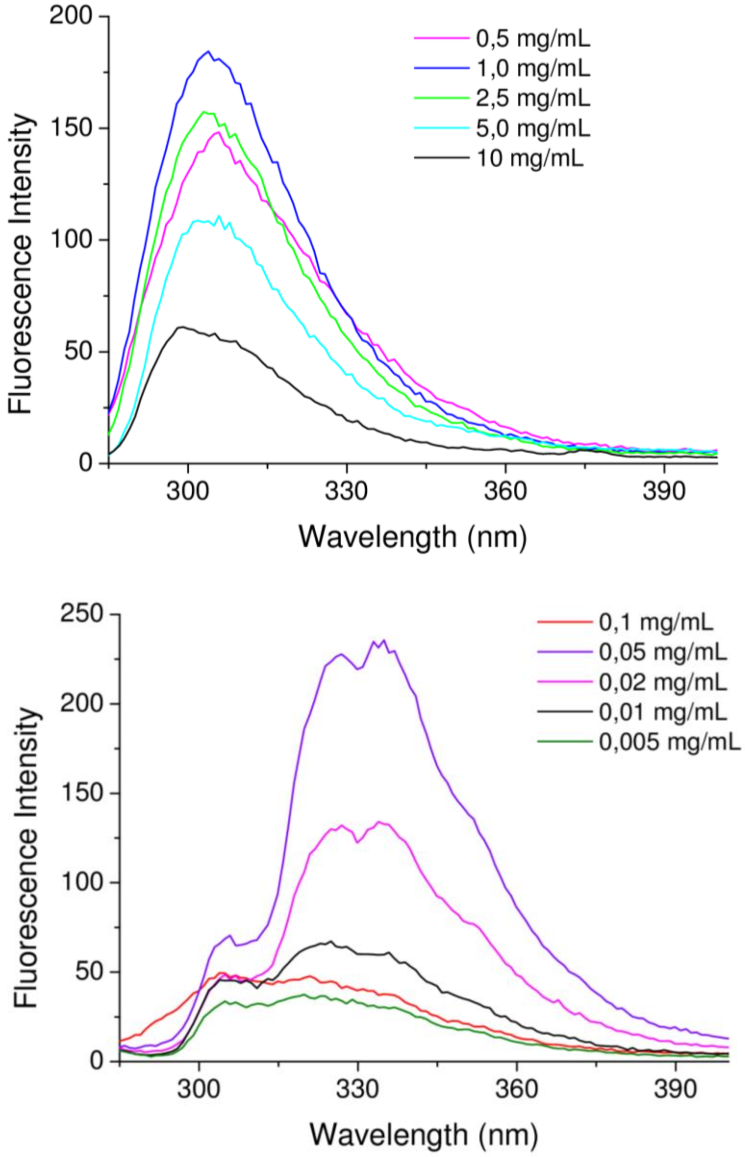


**Figure S10:** a) Fluorescence spectra of the peptide PEG8-FYFCFYF as function of the concentration between 0.5 and 10 mg/mL (a) and between 0.005 and 0.1 mg/mL (b) Excitation wavelength λ=17 nm and λ=275.

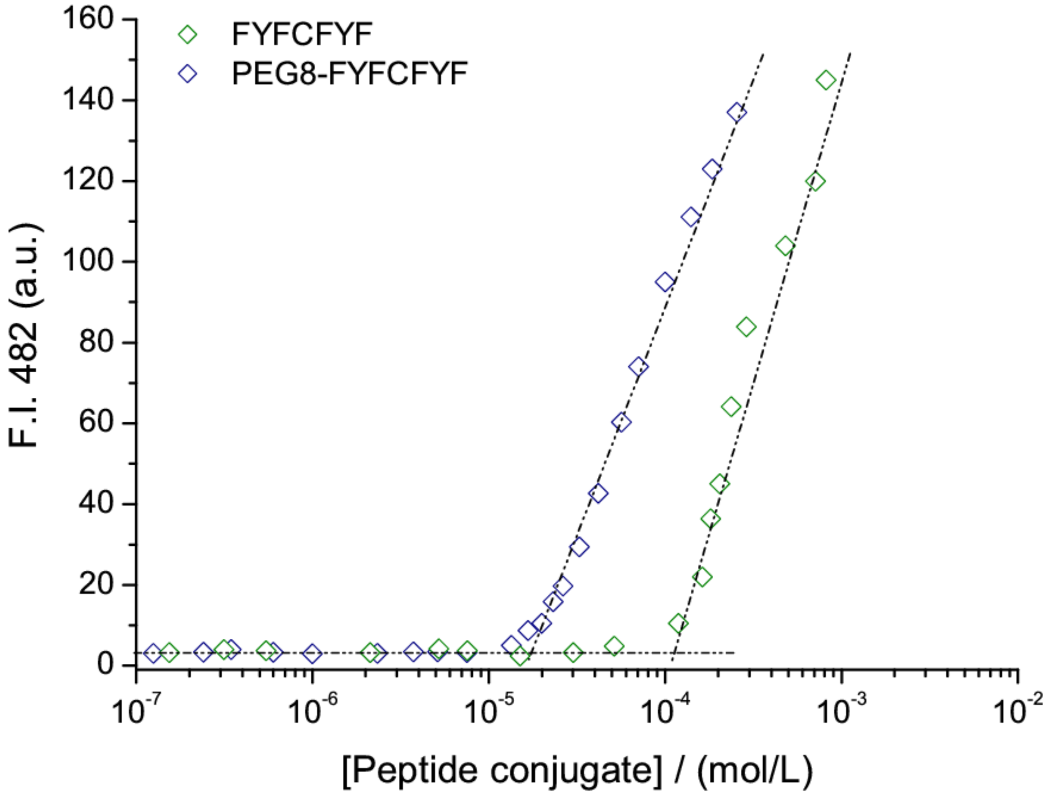


**Figure S11:** ThT titration (200µL, 50µmol/L) for estimation of CAC values from the graphical break point.

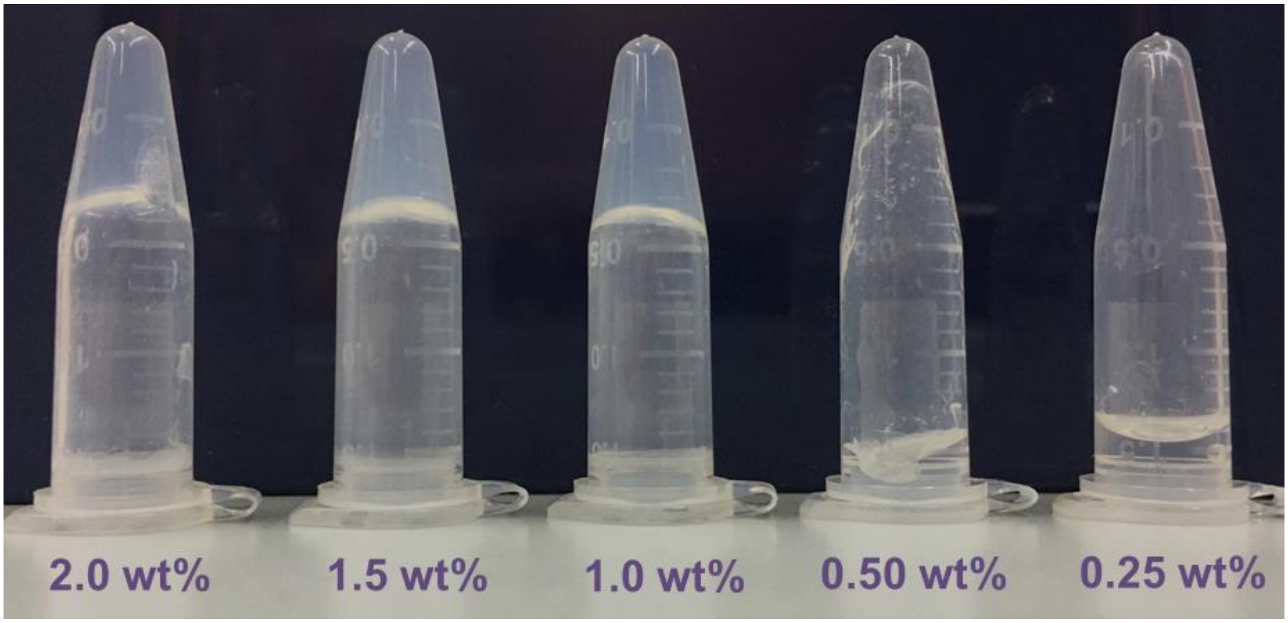


**Figure S12.** Determination of critical gelation concentration for FYFCFYF *via* inverted test tube.

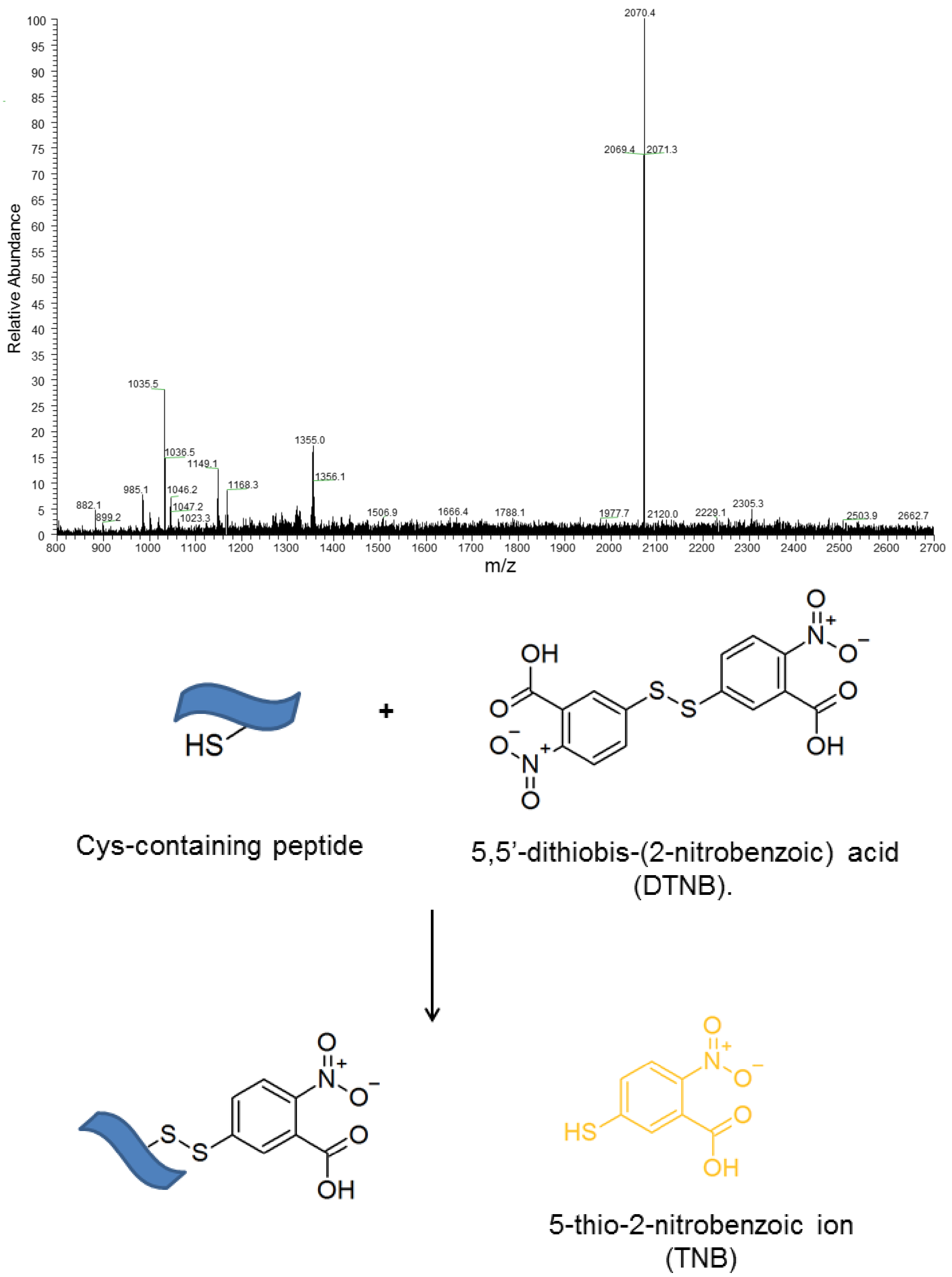


**Figure S13:** a) ESI mass spectrum of the oxidized peptide; b) the mechanism of reaction between DTNB and the Cys-containing peptide.

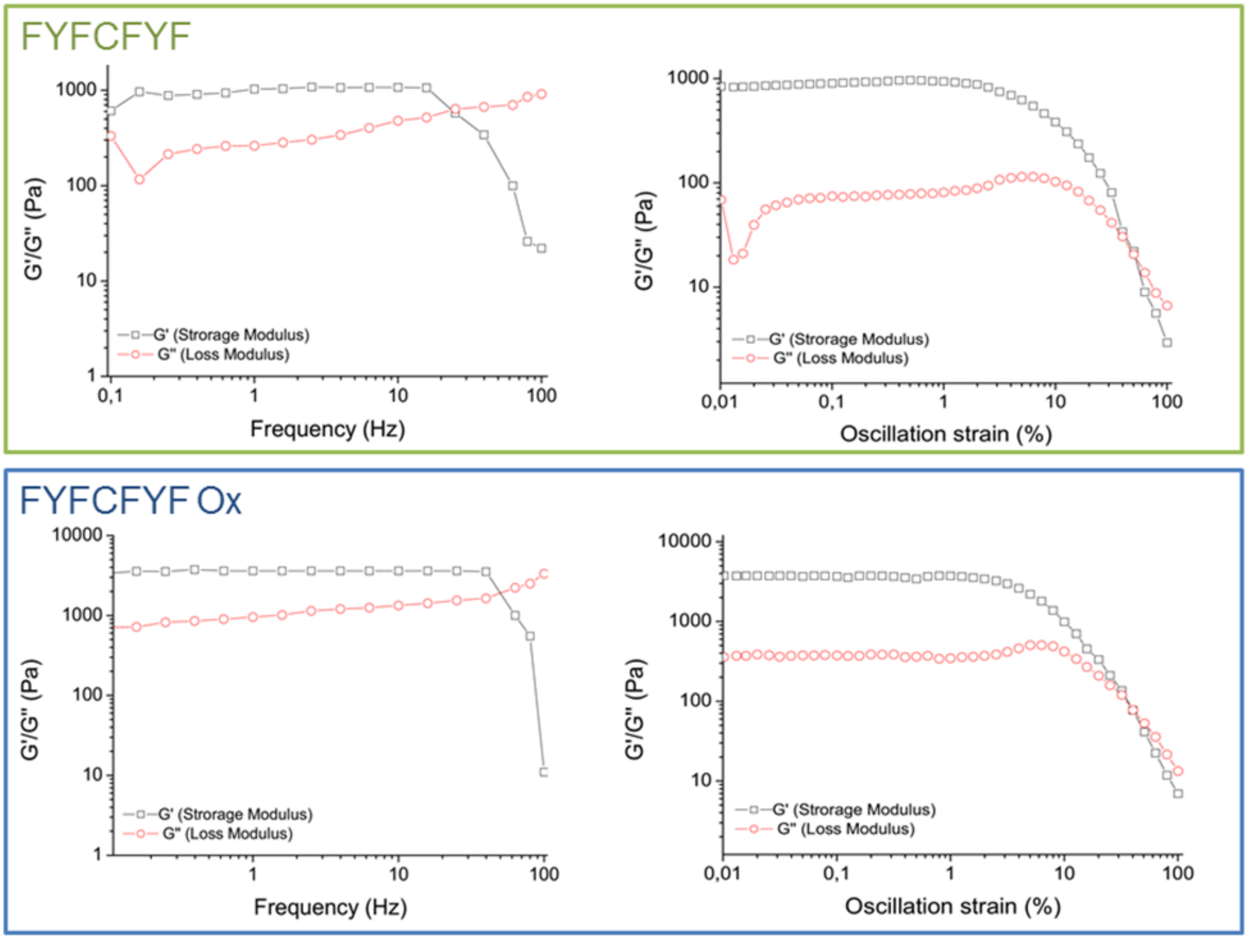


**Figure S14:** Dynamic frequency sweep oscillatory test at 0.1 % strain and dynamic strain sweep oscillatory test performed at 1 Hz frequency for FYFCFYF (green panel) and for FYFCFYF oxidized (blue panel) *via* AmBic 6.0 mmol/L.
